# CLUSS: Clustering of protein sequences based on a new similarity measure

**DOI:** 10.1186/1471-2105-8-286

**Published:** 2007-08-04

**Authors:** Abdellali Kelil, Shengrui Wang, Ryszard Brzezinski, Alain Fleury

**Affiliations:** 1 Département d'informatique, Faculté des Sciences, Université de Sherbrooke, Sherbrooke, QC, Canada; 2 Département de Biologie, Faculté des Sciences, Université de Sherbrooke, Sherbrooke, QC, Canada

## Abstract

**Background:**

The rapid burgeoning of available protein data makes the use of clustering within families of proteins increasingly important. The challenge is to identify subfamilies of evolutionarily related sequences. This identification reveals phylogenetic relationships, which provide prior knowledge to help researchers understand biological phenomena. A good evolutionary model is essential to achieve a clustering that reflects the biological reality, and an accurate estimate of protein sequence similarity is crucial to the building of such a model. Most existing algorithms estimate this similarity using techniques that are not necessarily biologically plausible, especially for hard-to-align sequences such as proteins with different domain structures, which cause many difficulties for the alignment-dependent algorithms. In this paper, we propose a novel similarity measure based on matching amino acid subsequences. This measure, named SMS for Substitution Matching Similarity, is especially designed for application to non-aligned protein sequences. It allows us to develop a new alignment-free algorithm, named CLUSS, for clustering protein families. To the best of our knowledge, this is the first alignment-free algorithm for clustering protein sequences. Unlike other clustering algorithms, CLUSS is effective on both alignable and non-alignable protein families. In the rest of the paper, we use the term "*phylogenetic*" in the sense of "*relatedness of biological functions*".

**Results:**

To show the effectiveness of CLUSS, we performed an extensive clustering on COG database. To demonstrate its ability to deal with hard-to-align sequences, we tested it on the GH2 family. In addition, we carried out experimental comparisons of CLUSS with a variety of mainstream algorithms. These comparisons were made on hard-to-align and easy-to-align protein sequences. The results of these experiments show the superiority of CLUSS in yielding clusters of proteins with similar functional activity.

**Conclusion:**

We have developed an effective method and tool for clustering protein sequences to meet the needs of biologists in terms of phylogenetic analysis and prediction of biological functions. Compared to existing clustering methods, CLUSS more accurately highlights the functional characteristics of the clustered families. It provides biologists with a new and plausible instrument for the analysis of protein sequences, especially those that cause problems for the alignment-dependent algorithms.

## Background

With the rapid burgeoning of protein sequence data, the number of proteins for which no experimental data are available greatly exceeds the number of functionally characterized proteins. To predict a function for an uncharacterized protein, it is necessary not only to detect its similarities to proteins of known biochemical properties (i.e., to assign the unknown protein to a family), but also to adequately assess the differences in cases where similar proteins have different functions (i.e., to distinguish among subfamilies). One solution is to cluster each family into distinct subfamilies composed of functionally related proteins. Subfamilies resulting from clustering are easier to analyze experimentally. A subfamily member that attracts particular interest need be compared only with the members of the same subfamily. A biological function can be attributed with high confidence to an uncharacterized protein, if a well-characterized protein within the same cluster is already known. Conversely, a biological function discovered for a newly characterized protein can be extended over all members of the same subfamily. In the rest of the paper, we use the terms subfamily and cluster interchangeably.

The literature reports many algorithms that can be used to build protein clustering databases, such as the widely used algorithm BLAST [1] and its improved versions Gapped-BLAST and PSI-BLAST [2], as well as SYSTERS [3], ProtClust [4] and ProtoMap [5] (see [6] for a review). These algorithms have been designed to deal with large sets of proteins by using various techniques to accelerate examination of the relationships between proteins. However, they are not very sensitive to the subtle differences among similar proteins. Consequently, these algorithms are not effective for clustering protein sequences in closely related families. On the other hand, more specific algorithms have also been developed, for instance, the widely cited algorithms BlastClust [7], which uses score-based single-linkage clustering, TRIBE-MCL [8], based on the Markov cluster approach, and gSPC [9], based on a method that is analogous to the treatment of an inhomogeneous ferromagnet in physics, as well as others such as those introduced by Sjölander [10], Wicker *et al*. [11] and Jothi *et al*. [12]. Almost all of these algorithms are either based on sequence alignment or rely on alignment-dependent algorithms for computing similarity. As several alignments are often possible for a single family, particularly for families which have not yet been definitively aligned and biologically approved, this will result in different clusterings. Such variable results create ambiguities and make biological interpretation of sequences a difficult task.

In this paper, we propose an efficient algorithm, CLUSS, for clustering protein families based on SMS, which is a new measure we propose for protein similarity. The novelty of CLUSS resides essentially in two features. First, CLUSS is applied directly to non-aligned sequences, thus eliminating the need for sequence pre-alignment. Second, it adopts a new measure of similarity, directly exploiting the substitution matrices generally used to align protein sequences and showing a great sensitivity to the relations among similar and divergent protein sequences. CLUSS can be summarized as follows (a detailed description of the algorithm is given later in the paper):

Given *F*, a family containing a given number of proteins:

**1) **Build a pairwise similarity matrix for the proteins in *F *using SMS our new similarity measure.

**2) **Create a phylogenetic tree of the protein family *F *using a hierarchical clustering approach.

**3) **Assign a co-similarity value to each node of the phylogenetic tree by applying a variant of Ward's formulas [13,14] introduced by Batagelj [15].

**4) **Calculate a critical threshold for identifying subfamily branches, by computing the interclass inertia [16].

**5) **Collect each leaf from its subfamily branch into a distinct subfamily (i.e., cluster).

## Implementation

CLUSS was developed with standard C++, and tested in a basic desktop computer under Microsoft Windows XP. The source code, the application server, and all experimental results are available at CLUSS website.

### The new similarity measure SMS

Many approaches to measuring the similarity between protein sequences have been developed. Prominent among these are alignment-dependent approaches including the well-known algorithm BLAST [1] and its improved versions Gapped-BLAST and PSI-BLAST [2], which the programs are available at [7], as well as several others such as the one introduced by Varré *et al*. [17] based on movements of segments, and the recent algorithm Scoredist introduced by Sonnhammer *et al*. [18] based on the logarithmic correction of observed divergence. These approaches often suffer from accuracy problems, especially for multi-domain, as well as circular permutation and tandem repeats protein sequences, which were well discussed by Higgins [19]. The similarity measures used in these approaches depend heavily on the quality of the alignment, which in turn depends on the alignability of the protein sequences. In many cases, alignment-free approaches can greatly improve protein comparison, especially for non-alignable protein sequences. These approaches have been reviewed in detail by several authors [20-23]. Their major drawback, in our opinion, is that they consider only the frequencies and lengths of similar regions within proteins and do not take into account the biological relationships that exist between amino acids. To correct this problem, some authors [22] have suggested the use of the Kimura correction method [24] or other types of corrections, such as that of Felsenstein [25]. However, to obtain an acceptable phylogenetic tree, the approach described in [22] performs an iterative refinement including a profile-profile alignment at each iteration, which significantly increases its complexity. Considering this, we have developed a new approach mainly motivated by biological considerations and known observations related to protein structure and evolution. The goal is to make efficient use of the information contained in amino acid subsequences in the proteins, which leads to a better similarity measurement. The principal idea of this approach is to use a substitution matrix such as BLOSUM62 [26] or PAM250 [27] to measure the similarity between matched amino acids from the protein sequences being compared.

**Figure 1:**
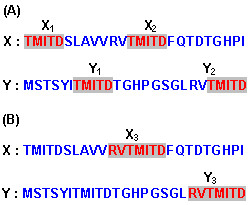
**Matching sequences**. Let *X *and *Y *be two protein sequences, as illustrated in figures **A **and **B**. **(A)**. For the pair of subsequences *x*_*1 *_and *y*_*1 *_we add a matching subsequence *Γ*_*1*_, identical to *x*_*1 *_and *y*_*1*_, to the matching set *E*^*4*^_*X*, *Y*_. Similarly, we add *Γ*_*2 *_identical to *x*_*1 *_and *y*_*2*_, and *Γ*_*3 *_identical to *x*_*2 *_and *y*_*1*_. However, since *x*_*2 *_⊂ *x*_*3 *_and *y*_*2 *_⊂ *y*_*3*_, (*x*_*3 *_and *y*_*3 *_are shown in figure **B**) we do not add *Γ*_*4*_, identical to *x*_*2 *_and *y*_*2*_, to *E*^*4*^_*X*, *Y*_. **(B)**. For the pair of subsequences *x*_*3 *_and *y*_*3 *_we add a matching subsequence *Γ*_*5*_, identical to *x*_*3 *_and *y*_*3*_, to the set *E*^*4*^_*X*, *Y*_, even if *x*_*3 *_overlaps with *x*_*2*_.

In this section, we will use the symbol |·| to express the length of a sequence. Let *X *and *Y *be two protein sequences belonging to the protein family *F*. Let *x *and *y *be two identical subsequences belonging respectively to *X *and *Y*; we use *Γ*_*x*, *y *_to represent the matched subsequence of *x *and *y*. We use *l *to represent the minimum length that *Γ*_*x*, *y *_should have; i.e., we will be interested only in *Γ*_*x*, *y *_whose length is at least *l *residues. We define *E*^*l*^_*X*, *Y*_, the key set of matched subsequences *Γ*_*x*, *y *_for the definition of our similarity function, as follows (see Figure 1 for an example):

EX,Yl={Γx,y||Γx,y|≥l,(∀Γx′,y′∈EX,Yl)∧(Γx′,y′≠Γx,y)⇒(x′⊄x)∨(y′⊄y)}
 MathType@MTEF@5@5@+=feaafiart1ev1aaatCvAUfKttLearuWrP9MDH5MBPbIqV92AaeXatLxBI9gBaebbnrfifHhDYfgasaacH8akY=wiFfYdH8Gipec8Eeeu0xXdbba9frFj0=OqFfea0dXdd9vqai=hGuQ8kuc9pgc9s8qqaq=dirpe0xb9q8qiLsFr0=vr0=vr0dc8meaabaqaciaacaGaaeqabaqabeGadaaakeaacqWGfbqrdaqhaaWcbaGaemiwaGLaeiilaWIaemywaKfabaGaemiBaWgaaOGaeyypa0ZaaiWabeaaiiGacqWFtoWrdaWgaaWcbaGaemiEaGNaeiilaWIaemyEaKhabeaakmaaeeaabaqbaeqabiqaaaqaamaaemaabaGae83KdC0aaSbaaSqaaiabdIha4jabcYcaSiabdMha5bqabaaakiaawEa7caGLiWoacqGHLjYScqWGSbaBcqGGSaalaeaadaqadaqaaiabgcGiIiab=n5ahnaaBaaaleaacuWG4baEgaqbaiabcYcaSiqbdMha5zaafaaabeaakiabgIGiolabdweafnaaDaaaleaacqWGybawcqGGSaalcqWGzbqwaeaacqWGSbaBaaaakiaawIcacaGLPaaacqGHNis2daqadaqaaiab=n5ahnaaBaaaleaacuWG4baEgaqbaiabcYcaSiqbdMha5zaafaaabeaakiabgcMi5kab=n5ahnaaBaaaleaacqWG4baEcqGGSaalcqWG5bqEaeqaaaGccaGLOaGaayzkaaGaeyO0H49aaeWaaeaacuWG4baEgaqbaiabgsOillabdIha4bGaayjkaiaawMcaaiabgIIiApaabmaabaGafmyEaKNbauaacqGHekYYcqWG5bqEaiaawIcacaGLPaaaaaaacaGLhWoaaiaawUhacaGL9baaaaa@79F8@

The symbols *x' *and *y' *in the formula are simply used as variables in the same way as *x *and *y*. The expression (. ⊄ .) means that the first element is not included in the second one, either in terms of the composition of the subsequences or in terms of their respective positions in *X*. The matching set *E*^*l*^_*X*, *Y *_contains all the matched subsequences of maximal length between the sequences *X *and *Y*. It will be used to compute the matching score of the sequence pair.

The formula *E*^*l*^_*X*, *Y *_adequately describes some known properties of polypeptides and proteins. First, protein motifs (i.e., series of defined residues) determine the tendency of the primary structure to adopt a particular secondary structure, a property exploited by several secondary-structure prediction algorithms. Such motifs can be as short as four residues (for instance those found in *β*-turns), but the propensity to form an *α*-helix or a *β*-sheet is usually defined by longer motifs. Second, our proposal to take into account multiple (i.e., ≥2) occurrences of a particular motif reflects the fact that sequence duplication is one of the most powerful mechanisms of gene and protein evolution, and if a motif is found twice (or more) in a protein it is more probable that it was acquired by duplication of a segment from a common ancestor than by acquisition from a distant ancestor. The following pseudo-code describes how we can obtain the matching set *E*^*l*^_*X*, *Y*_:

*Γ*: matched subsequence.

*E*: matching set.

**for **i = 1 to maximum of |*X*| **and **|*Y*|

   k = 0, j = i

   **while **(k < |*X*| **and **j < |*Y*|)

      **if **(*X*[k] = *Y *[j])

      **then **Add the amino acid *X*[k] to *Γ*

      **else **If (|*Γ*| ≥ *l*) Add the *Γ *to *E*

         Empty *Γ*

      **end else**

      Increment k, Increment j

   **end while**

   **if **(|*Γ*| ≥ *l*) Add *Γ *to *E*

   Empty *Γ*

   k = i, j = 0

   **while **(k < |*X*| **and **j < |*Y*|)

      **if **(*X*[k] = *Y*[j])

      **then **Add the amino acid *X*[k] to *Γ*

      **else **if (|*Γ*| ≥ *l*) Add *Γ *to *E*

         Empty *Γ*

      **end else**

      Increment k, Increment j

   **end while**

   **if **(|*Γ*| ≥ *l*) Add *Γ *to *E*

end for

This algorithm for the construction of *E*^*l*^_*X*, *Y *_requires a CPU time proportional to |*X*|*|*Y*|. In practice, however, several optimizations are possible in the implementation, using encoding techniques to speed up this process. In our implementation of SMS, we used a technique that improved considerably the speed of the algorithm; we can summarize it as follows:

By the property that all possible matched subsequences satisfy |*Γ*_*x, y*_|≥*l*, we know that each *Γ*_*x*, *y *_in *E*^*l*^_*X*, *Y *_is an expansion of a matched subsequence of length *l*. Thus, we first collect all the matched subsequences of length *l*, which takes linear time. Secondly, we expand each of the matched subsequences as much as possible on the both left and right sides. Finally, we select all the expanded matched sequences that are maximal according to the inclusion criterion. This technique is very efficient for reducing the execution time in practice. However, due to the variable lengths of the matched sequences, it may not be possible to reduce the worst-case complexity to a linear time. In the Results section, we provide a time comparison between our algorithm and several existing ones.

Let *M *be a substitution matrix, and *Γ *a matched subsequence belonging to the matching set *E*^*l*^_*X*, *Y*_. We define a weight *W*(*Γ*) for the matched subsequence *Γ*, to quantify its importance compared to all the other subsequences of *E*^*l*^_*X*, *Y*_, as follows:

W(Γ)=∑i=1|Γ|M[Γ[i],Γ[i]]
 MathType@MTEF@5@5@+=feaafiart1ev1aaatCvAUfKttLearuWrP9MDH5MBPbIqV92AaeXatLxBI9gBaebbnrfifHhDYfgasaacH8akY=wiFfYdH8Gipec8Eeeu0xXdbba9frFj0=OqFfea0dXdd9vqai=hGuQ8kuc9pgc9s8qqaq=dirpe0xb9q8qiLsFr0=vr0=vr0dc8meaabaqaciaacaGaaeqabaqabeGadaaakeaacqWGxbWvdaqadaqaaGGaciab=n5ahbGaayjkaiaawMcaaiabg2da9maaqahabaGaemyta00aamWaaeaacqWFtoWrdaWadaqaaiabdMgaPbGaay5waiaaw2faaiabcYcaSiab=n5ahnaadmaabaGaemyAaKgacaGLBbGaayzxaaaacaGLBbGaayzxaaaaleaacqWGPbqAcqGH9aqpcqaIXaqmaeaadaabdaqaaiab=n5ahbGaay5bSlaawIa7aaqdcqGHris5aaaa@494A@

Where *Γ*[*i*] is the *i*^*th *^amino acid of the matched subsequence *Γ*, and *W*[*Γ*[*i*], *Γ*[*i*]] is the substitution score of this amino acid with itself. Here, in order to make our measure biologically plausible, we use the substitution concept to emphasize the relation that binds one amino acid with itself. The value of *M*[*Γ*[*i*], *Γ*[*i*]] (i.e., within the diagonal of the substitution matrix) estimate the rate at which each possible amino acid in a sequence keep unchanged over time. For the pair of sequences *X *and *Y*, we define the matching score *s*_*X*, *Y*_, understood as representing the substitution relation of the conserved regions in both sequences, as follows:

sX,Y=∑Γ∈EX,YlW(Γ)MAX(|X|,|Y|)
 MathType@MTEF@5@5@+=feaafiart1ev1aaatCvAUfKttLearuWrP9MDH5MBPbIqV92AaeXatLxBI9gBaebbnrfifHhDYfgasaacH8akY=wiFfYdH8Gipec8Eeeu0xXdbba9frFj0=OqFfea0dXdd9vqai=hGuQ8kuc9pgc9s8qqaq=dirpe0xb9q8qiLsFr0=vr0=vr0dc8meaabaqaciaacaGaaeqabaqabeGadaaakeaacqWGZbWCdaWgaaWcbaGaemiwaGLaeiilaWIaemywaKfabeaakiabg2da9maalaaabaWaaabuaeaacqWGxbWvdaqadaqaaGGaciab=n5ahbGaayjkaiaawMcaaaWcbaGae83KdCKaeyicI4Saemyrau0aa0baaWqaaiabdIfayjabcYcaSiabdMfazbqaaiabdYgaSbaaaSqab0GaeyyeIuoaaOqaaiabd2eanjabdgeabjabdIfayjabcIcaOmaaemaabaGaemiwaGfacaGLhWUaayjcSdGaeiilaWYaaqWaaeaacqWGzbqwaiaawEa7caGLiWoacqGGPaqkaaaaaa@50AE@

To define our similarity measure, we need to scale down *s*_*X*, *Y*_. Let *s*_*max *_be the matching score of the longest sequence belonging to the protein family *F *with itself, defined as follows:

*s*_max _= {*s*_*X*, *X*_;|*X*| = max {|*Y*|;*Y *⊂ *F*}}

Finally, the similarity measure between the two sequences *X *and *Y*, *S*_*X*, *Y *_is obtained by dividing the matching score by the value of *s*_*max*_:

SX,Y=sX,Ysmax⁡
 MathType@MTEF@5@5@+=feaafiart1ev1aaatCvAUfKttLearuWrP9MDH5MBPbIqV92AaeXatLxBI9gBaebbnrfifHhDYfgasaacH8akY=wiFfYdH8Gipec8Eeeu0xXdbba9frFj0=OqFfea0dXdd9vqai=hGuQ8kuc9pgc9s8qqaq=dirpe0xb9q8qiLsFr0=vr0=vr0dc8meaabaqaciaacaGaaeqabaqabeGadaaakeaacqWGtbWudaWgaaWcbaGaemiwaGLaeiilaWIaemywaKfabeaakiabg2da9maalaaabaGaem4Cam3aaSbaaSqaaiabdIfayjabcYcaSiabdMfazbqabaaakeaacqWGZbWCdaWgaaWcbaGagiyBa0MaeiyyaeMaeiiEaGhabeaaaaaaaa@3D35@

### Minimum length of matched subsequences "*l*"

In the CLUSS algorithm described in the following section, *l*, the minimum length of the matched subsequences in SMS, is set to 4. *l *= 4 yields good results in all our experiments. Here we will attempt to provide an explanation of this choice.

Our aim is to detect and make use of the significant motifs best conserved during evolution and to minimize the influence of those motifs that occur by chance. This motivates one of the major biological features of our similarity measure, the inclusion of all long conserved subsequences in the matching (i.e., multiple occurrences), since it is well known that the longer the subsequences, the smaller the chance of their being identical by chance, and vice-versa. Here we make use of the theory developed by Karlin *et al*. in [28-30] to justify our choice of *l*. According to theorem 1 in [29] we have:

Kr,N=log⁡n(|Seq1|,...,|SeqN|)+log⁡λ(1−λ)+0.577−log⁡λ
 MathType@MTEF@5@5@+=feaafiart1ev1aaatCvAUfKttLearuWrP9MDH5MBPbIqV92AaeXatLxBI9gBaebbnrfifHhDYfgasaacH8akY=wiFfYdH8Gipec8Eeeu0xXdbba9frFj0=OqFfea0dXdd9vqai=hGuQ8kuc9pgc9s8qqaq=dirpe0xb9q8qiLsFr0=vr0=vr0dc8meaabaqaciaacaGaaeqabaqabeGadaaakeaacqWGlbWsdaWgaaWcbaGaemOCaiNaeiilaWIaemOta4eabeaakiabg2da9maalaaabaGagiiBaWMaei4Ba8Maei4zaCMaemOBa42aaeWaaeaadaabdaqaaiabdofatjabdwgaLjabdghaXnaaBaaaleaacqaIXaqmaeqaaaGccaGLhWUaayjcSdGaeiilaWIaeiOla4IaeiOla4IaeiOla4IaeiilaWYaaqWaaeaacqWGtbWucqWGLbqzcqWGXbqCdaWgaaWcbaGaemOta4eabeaaaOGaay5bSlaawIa7aaGaayjkaiaawMcaaiabgUcaRiGbcYgaSjabc+gaVjabcEgaNHGaciab=T7aSnaabmaabaGaeGymaeJaeyOeI0Iae83UdWgacaGLOaGaayzkaaGaey4kaSIaeGimaaJaeiOla4IaeGynauJaeG4naCJaeG4naCdabaGaeyOeI0IagiiBaWMaei4Ba8Maei4zaCMae83UdWgaaaaa@66CE@

where

n(|Seq1|,...,|SeqN|)=∑1≤i1≤...≤ir≤N∏ν=1r|Seqiν|
 MathType@MTEF@5@5@+=feaafiart1ev1aaatCvAUfKttLearuWrP9MDH5MBPbIqV92AaeXatLxBI9gBaebbnrfifHhDYfgasaacH8akY=wiFfYdH8Gipec8Eeeu0xXdbba9frFj0=OqFfea0dXdd9vqai=hGuQ8kuc9pgc9s8qqaq=dirpe0xb9q8qiLsFr0=vr0=vr0dc8meaabaqaciaacaGaaeqabaqabeGadaaakeaacqWGUbGBdaqadaqaamaaemaabaGaem4uamLaemyzauMaemyCae3aaSbaaSqaaiabigdaXaqabaaakiaawEa7caGLiWoacqGGSaalcqGGUaGlcqGGUaGlcqGGUaGlcqGGSaaldaabdaqaaiabdofatjabdwgaLjabdghaXnaaBaaaleaacqWGobGtaeqaaaGccaGLhWUaayjcSdaacaGLOaGaayzkaaGaeyypa0ZaaabuaeaadaqeWbqaamaaemaabaGaem4uamLaemyzauMaemyCae3aaSbaaSqaaiabdMgaPnaaBaaameaaiiGacqWF9oGBaeqaaaWcbeaaaOGaay5bSlaawIa7aaWcbaGae8xVd4Maeyypa0JaeGymaedabaGaemOCaihaniabg+GivdaaleaacqaIXaqmcqGHKjYOcqWGPbqAdaWgaaadbaGaeGymaedabeaaliabgsMiJkabc6caUiabc6caUiabc6caUiabgsMiJkabdMgaPnaaBaaameaacqWGYbGCaeqaaSGaeyizImQaemOta4eabeqdcqGHris5aaaa@6AC4@

and

λ=max⁡1≤ν1≤...≤νr≤N(∑i=120∏j=1rpi(νj))
 MathType@MTEF@5@5@+=feaafiart1ev1aaatCvAUfKttLearuWrP9MDH5MBPbIqV92AaeXatLxBI9gBaebbnrfifHhDYfgasaacH8akY=wiFfYdH8Gipec8Eeeu0xXdbba9frFj0=OqFfea0dXdd9vqai=hGuQ8kuc9pgc9s8qqaq=dirpe0xb9q8qiLsFr0=vr0=vr0dc8meaabaqaciaacaGaaeqabaqabeGadaaakeaaiiGacqWF7oaBcqGH9aqpdaWfqaqaaiGbc2gaTjabcggaHjabcIha4bWcbaGaeGymaeJaeyizImQae8xVd42aaSbaaWqaaiabigdaXaqabaWccqGHKjYOcqGGUaGlcqGGUaGlcqGGUaGlcqGHKjYOcqWF9oGBdaWgaaadbaGaemOCaihabeaaliabgsMiJkabd6eaobqabaGcdaqadaqaamaaqahabaWaaebCaeaacqWGWbaCdaqhaaWcbaGaemyAaKgabaGaeiikaGIae8xVd42aaSbaaWqaaiabdQgaQbqabaWccqGGPaqkaaaabaGaemOAaOMaeyypa0JaeGymaedabaGaemOCaihaniabg+GivdaaleaacqWGPbqAcqGH9aqpcqaIXaqmaeaacqaIYaGmcqaIWaama0GaeyyeIuoaaOGaayjkaiaawMcaaaaa@5D75@

These formulas calculates *K*_*r, N*_, the *expected length of the longest common word present in at least r out of N sequences *[29] (i.e., *Seq*_*1*_,...,*Seq*_*N*_), where *p*_*i*_^(*ν*) ^is generally specified as the *i*^*th *^residue frequency of the observed *ν*^(*th*) ^sequence.

By fixing *N *= *r *= 2, we calculated *K*_2,2_, the expected length of the longest matched subsequence present by chance at least 2 times out of each pair of sequences, for several protein datasets including the COG [31] database and the G-proteins [32], GH2 [33] and ROK [34] families. The results, presented in Table 1, show an average expected length very close to *K*_2,2 _= 4 residues, with a relatively small standard deviation for each dataset. Thus, for lengths equal to or greater than four amino acids, identical protein subsequences are more likely to be conserved motifs. This choice of length was also made in previous protein sequence comparison contexts, such as Heringa [35] for secondary structure prediction and Leung *et al*. [36] for identifying matches in multiple long sequences.

**Table 1:** Expected length of longest common subsequence computed for several protein datasets. The columns represent respectively, DS: the tested protein datasets, NS: number of tested protein sequences, AEL: average of the expected length of the longest common subsequence and finally SD: the standard deviation.

DS	NS	AEL	SD
COG database	144298	3.934	0.363
KOG database	60748	4.062	0.458
G-proteins family	381	3.718	0.200
GH2 family	316	4.355	0.232
ROK family	730	4.074	0.324

### The CLUSS algorithm

CLUSS is composed of three main stages. The first one consists in building a pairwise similarity matrix based on our new similarity measure SMS; the second, in building a phylogenetic tree according to the similarity matrix, using a hierarchical approach; and the third, in identifying subfamily nodes from which leaves are grouped into subfamilies.

### Stage 1: Similarity matrix

Using one of the known substitution score matrices, such as BLOSUM62 [26] or PAM250 [27], and our new similarity measure, we compute *S*, the (*N *× *N*) pairwise similarity matrix, where *N *is the number of sequences of the protein family *F *to be clustered, and *S*_*i, j *_is the similarity measure between the *i*^*th *^and the *j*^*th *^protein sequences belonging to *F*. The construction of *S *takes CPU time proportional to *N*(*N-1*)*T*^2^/*2*, with *T *the typical sequence length of the *N *sequences.

### Stage 2: Phylogenetic tree

To build the phylogenetic tree, we have adopted the classical hierarchical approach. Starting from the protein sequences, each of which is considered as the root node of a (sub)tree containing only one node, we iteratively join a pair of root nodes in order to build a bigger subtree. At each iteration, a pair of root nodes is selected if they are the most similar root nodes in terms of a similarity measure derived from the above similarity matrix *S*. This process ends when there remains only one (sub)tree, which is the phylogenetic tree.

The similarity between two root nodes referred to above is computed in the following way. At the beginning of the iteration, the similarity between any pair of nodes is initialized by the similarity matrix computed in **Stage 1 **(i.e., according to SMS). Let *L *and *R *be two nearest root nodes at a given iteration step; they are joined together to form a new subtree. Let *P *be the root node of the new subtree. *P *thus has two children, *L *and *R*. We assign a "length" value *D*_*L, P *_= *D*_*R, P *_= (*1*-*S*_*L, R*_)/2 to each of the two branches connecting *L *and *R *to *P*. This value is the estimate of the phylogenetic distance from either node *L *or *R *to their parent *P *in the tree. This distance has no strict mathematical sense; it is merely a measure of the evolutionary distance between the nodes. It is closer to the notion of dissimilarity. The similarity between the new root node *P *and any other root node *K *is defined as a weighted average of the similarity between the children of *P *and the node *K*:

SP,K=dL∗SL,K+dR∗SR,KdL+dR
 MathType@MTEF@5@5@+=feaafiart1ev1aaatCvAUfKttLearuWrP9MDH5MBPbIqV92AaeXatLxBI9gBaebbnrfifHhDYfgasaacH8akY=wiFfYdH8Gipec8Eeeu0xXdbba9frFj0=OqFfea0dXdd9vqai=hGuQ8kuc9pgc9s8qqaq=dirpe0xb9q8qiLsFr0=vr0=vr0dc8meaabaqaciaacaGaaeqabaqabeGadaaakeaacqWGtbWudaWgaaWcbaGaemiuaaLaeiilaWIaem4saSeabeaakiabg2da9maalaaabaGaemizaq2aaSbaaSqaaiabdYeambqabaGccqGHxiIkcqWGtbWudaWgaaWcbaGaemitaWKaeiilaWIaem4saSeabeaakiabgUcaRiabdsgaKnaaBaaaleaacqWGsbGuaeqaaOGaey4fIOIaem4uam1aaSbaaSqaaiabdkfasjabcYcaSiabdUealbqabaaakeaacqWGKbazdaWgaaWcbaGaemitaWeabeaakiabgUcaRiabdsgaKnaaBaaaleaacqWGsbGuaeqaaaaaaaa@49B5@

Where *S*_*L*, *K *_and *S*_*R*, *K *_are in that order the similarity values between the nodes *L *and *R *with the node *K *before the joining, and *d*_*L *_and *d*_*R *_are the numbers of leaves in the subtree rooted at *L *and *R*, respectively. Note that in order to keep the notation simple, *S*_*P*, *K *_is retained here to represent the similarity between any pair of nodes that do not have any descendant relationships in the phylogenetic tree.

### Stage 3: Clusters extraction

Given *F*, a family of *N *protein sequences, after computing their similarity matrix and phylogenetic tree, CLUSS locates subfamily nodes in this tree using [13,14] Ward's approach. The main idea is to extract from the phylogenetic tree a number of subtrees, each of which corresponds to a cluster, while optimizing a validation criterion. The criterion is in fact a trade-off between the within-cluster compactness and the between-cluster separation [16]. The different steps are summarized as follows:

#### Step 1 (Computing the weight of each node)

First, each leaf node is considered as a subtree in the phylogenetic tree. We assign to each subtree *L *(i.e., an individual leaf represents one protein sequence) a weight *W*_*L *_according to its importance in *F*. *W*_*L *_depends on the number and closeness of the protein sequences that are in fact similar to *L*, and is thus intended to measure how well *F *is represented by this particular sequence. For this purpose, we make use of the Thompson [37] method in the definition of *W*_*L*_:

WL=∑i∈{branch(L→P)−{P}}DParent(i)i,dParent(i)
 MathType@MTEF@5@5@+=feaafiart1ev1aaatCvAUfKttLearuWrP9MDH5MBPbIqV92AaeXatLxBI9gBaebbnrfifHhDYfgasaacH8akY=wiFfYdH8Gipec8Eeeu0xXdbba9frFj0=OqFfea0dXdd9vqai=hGuQ8kuc9pgc9s8qqaq=dirpe0xb9q8qiLsFr0=vr0=vr0dc8meaabaqaciaacaGaaeqabaqabeGadaaakeaacqWGxbWvdaWgaaWcbaGaemitaWeabeaakiabg2da9maaqafabaWaaSaaaeaacqWGebardaWgaaWcbaGaemiuaaLaemyyaeMaemOCaiNaemyzauMaemOBa4MaemiDaqNaeiikaGIaemyAaKMaeiykaKIaemyAaKMaeiilaWcabeaaaOqaaiabdsgaKnaaBaaaleaacqWGqbaucqWGHbqycqWGYbGCcqWGLbqzcqWGUbGBcqWG0baDcqGGOaakcqWGPbqAcqGGPaqkaeqaaaaaaeaacqWGPbqAcqGHiiIZdaGadeqaaiabdkgaIjabdkhaYjabdggaHjabd6gaUjabdogaJjabdIgaOnaabmaabaGaemitaWKaeyOKH4QaemiuaafacaGLOaGaayzkaaGaeyOeI0YaaiWabeaacqWGqbauaiaawUhacaGL9baaaiaawUhacaGL9baaaeqaniabggHiLdaaaa@64C0@

Where *P *is the root of the phylogenetic tree, *L *a leaf in this tree, *branch(L→P)*-{*P*} the subset of nodes on the branch from *L *to *P *excluding *P*, *Parent(i) *the parent of the node *i*, *D*_*Parent(i), i *_is the length of the branch connecting the node *i *to its parent (as defined in the previous phase), and *d*_*Parent(i) *_the number of leaves in the subtree rooted at the parent of *i*. According to this definition, the value of *W*_*L *_is small if *L *is very representative and is large if *L *is not very representative. Iteratively, we assign to each internal subtree *P *the weight value *W*_*P *_equal to the sum of the weights of its children *W*_*L *_+ *W*_*R*_.

#### Step 2 (Computing co-similarity for all internal nodes)

Iteratively, until the root of the phylogenetic tree is reached, we assign to the subtree rooted at each non-leaf node *P *the co-similarity value *C*_*P *_(between its two child nodes), which is calculated according to the generalized Ward dissimilarity formula [13,14] introduced by Batagelj [15], as follows:

CP=WL∗WRWL+WR∗SL,R
 MathType@MTEF@5@5@+=feaafiart1ev1aaatCvAUfKttLearuWrP9MDH5MBPbIqV92AaeXatLxBI9gBaebbnrfifHhDYfgasaacH8akY=wiFfYdH8Gipec8Eeeu0xXdbba9frFj0=OqFfea0dXdd9vqai=hGuQ8kuc9pgc9s8qqaq=dirpe0xb9q8qiLsFr0=vr0=vr0dc8meaabaqaciaacaGaaeqabaqabeGadaaakeaacqWGdbWqdaWgaaWcbaGaemiuaafabeaakiabg2da9maalaaabaGaem4vaC1aaSbaaSqaaiabdYeambqabaGccqGHxiIkcqWGxbWvdaWgaaWcbaGaemOuaifabeaaaOqaaiabdEfaxnaaBaaaleaacqWGmbataeqaaOGaey4kaSIaem4vaC1aaSbaaSqaaiabdkfasbqabaaaaOGaey4fIOIaem4uam1aaSbaaSqaaiabdYeamjabcYcaSiabdkfasbqabaaaaa@41C9@

Where *W*_*L *_and *W*_*R *_are the weights of *L *and *R*, respectively, and *S*_*L, R *_is the similarity between *L *and *R *computed in Stage 2.

By taking into account information about the neighbourhood around each of the nodes *L *and *R*, the concept of co-similarity reflects the cluster compactness of all the sequences (leaf nodes) in the subtree. In fact, its value is inversely proportional to the within-cluster variance. When the subtree becomes larger, the co-similarity tends to become smaller, which means that the sequences within the subtree become less similar and the difference (separation) between sequences in different clusters becomes less significant.

#### Step 3 (Separating high co-similarity nodes from low co-similarity nodes)

The CLUSS algorithm makes use of a systematic method for deciding which subtrees to retain as a trade-off between searching for the highest co-similarity values and searching for the largest possible clusters. We first separate all the subtrees into two groups, one being the group of high co-similarity subtrees and the other the low co-similarity subtrees. This is done by sorting all possible subtrees in increasing order of co-similarity and computing a separation threshold according to the method based on the maximum interclass inertia [11].

#### Step 4 (Extracting clusters)

**Figure 2:**
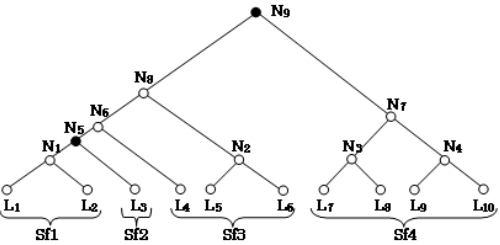
**Merging leaves**. Let us take a rooted phylogenetic tree with *L*_*1*_, *L*_*2*_...*L*_*10 *_as leaves, and *N*_*1*_, *N*_*2*_...*N*_*9 *_as internal nodes, where *N*_*5 *_and *N*_*9 *_are identified as low co-similarity nodes (black nodes). Leaves are merged until a black node is reached, except for *L*_*3*_, *L*_*4*_, *L*_*5 *_and *L*_*6*_, which need special consideration. All leaves connected between *N*_*5 *_and *N*_*9 *_are merged into a distinct subfamily. *L*_*3 *_is connected directly to *N*_*5 *_so it constitutes a distinct subfamily. We thus obtain the subfamilies *Sf1*, *Sf2*, *Sf3 *and *Sf4*, while *Sf2 *contains the orphan sequence represented by leaf *L3*.

From the group of high co-similarity subtrees, we extract those that are largest. A high co-similarity subtree is largest if the following two conditions are satisfied: 1) it does not contain any low co-similarity subtree; 2) if it is included in another high co-similarity subtree, the latter contains at least one low co-similarity subtree. Each of these (largest) subtrees corresponds to a cluster and its leaves are then collected to form the corresponding cluster (see Figure 2 for an example).

## Results

To illustrate its efficiency, we tested CLUSS extensively on a variety of protein datasets and databases and compared it with several mainstream clustering algorithms. We analyzed the results obtained for the different tests with support from the literature and functional annotations. Most important data and results are provided with this paper as supplementary material, the others are available at CLUSS Website.

### The clustering quality measure

To highlight the functional characteristics and classifications of the clustered families, we introduce the *Q-measure*, which quantifies the quality of a clustering by measuring the percentage of correctly clustered protein sequences based on their known functional annotations. This measure can be easily adapted to any protein sequence database. The *Q-measure *is defined as follows:

Q−measure=(∑i=1CPi)−UN⋅100
 MathType@MTEF@5@5@+=feaafiart1ev1aaatCvAUfKttLearuWrP9MDH5MBPbIqV92AaeXatLxBI9gBaebbnrfifHhDYfgasaacH8akY=wiFfYdH8Gipec8Eeeu0xXdbba9frFj0=OqFfea0dXdd9vqai=hGuQ8kuc9pgc9s8qqaq=dirpe0xb9q8qiLsFr0=vr0=vr0dc8meaabaqaciaacaGaaeqabaqabeGadaaakeaacqWGrbqucqGHsislcqWGTbqBcqWGLbqzcqWGHbqycqWGZbWCcqWG1bqDcqWGYbGCcqWGLbqzcqGH9aqpdaWcaaqaamaabmaabaWaaabCaeaacqWGqbaudaWgaaWcbaGaemyAaKgabeaaaeaacqWGPbqAcqGH9aqpcqaIXaqmaeaacqWGdbWqa0GaeyyeIuoaaOGaayjkaiaawMcaaiabgkHiTiabdwfavbqaaiabd6eaobaacqGHflY1cqaIXaqmcqaIWaamcqaIWaamaaa@4CB2@

Where *N *is the total number of clustered sequences, *C *is the number of clusters obtained, *P*_*i *_is the largest number of sequences in the *i*^*th *^cluster obtained belonging to the same function group according to the known reference classification, and *U *is the number of unclustered sequences. For the extreme case where each cluster contains one protein with all proteins classified as such, the *Q-measure *is 0, since *C *becomes equal to *N*, and each *P*_*i *_the largest number of obtained sequences in the *i*^*th *^cluster is 1.

### COG database

To illustrate the efficiency of CLUSS in grouping protein sequences according to their functional annotation and biological classification, we performed extensive tests on the phylogenetic classification of proteins encoded in complete genomes, commonly named the Clusters of Orthologous Groups of proteins database (COG) [31]. As mentioned in the website for the database, the COG clusters were delineated by comparing protein sequences encoded in complete genomes, representing major phylogenetic lineages. Each COG consists of individual proteins or groups of paralogs from at least 3 lineages and thus corresponds to an ancient conserved domain. In order to evaluate CLUSS in a statistical manner, we randomly generated 1000 different subsets from the COG database. Each subset contains between 59 and 1840 non-orphan protein sequences (i.e., each selected protein sequence has at least one similar protein sequence from the same functional classification of the COG database).

**Figure 3:**
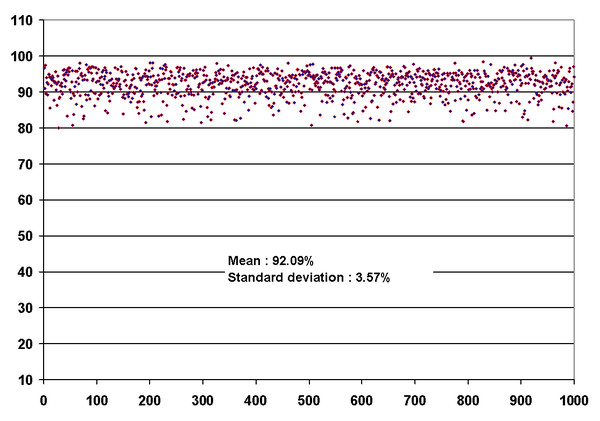
**Clustering results for the 1000 subsets from COG**. Each red point is a quality measure (*Q-measure*) of a clustering result of one of the 1000 randomly generated subsets from the COG database. As shown, the obtained results are in good concordance with the functional reference characterization of COG. The average of the quality measure of the 1000 clusterings is equal to **92.09% **with a standard deviation equal to **3.57%**. More than **75% **of the 1000 clusterings obtained a quality measure superior to **90%**, and more than **21% **of the clusterings obtained a quality measure superior to **95%**. The minimum value of the quality measure is **80.03% **and the maximum value is **99.35%**.

We tested CLUSS on the 1000 subsets using each of the substitution matrices BLOSUM62 [26] and PAM250 [27] to compute SMS. The average *Q-measure *value of the clusterings obtained is superior to **92% **with a standard deviation of **3.57% **(see Figure 3), while the minimum *Q-measure *value is **80.03% **and the maximum value is **99.35%**. This result shows that CLUSS is indeed very effective in grouping sequences according to the known functional classification of COG.

**Figure 4:**
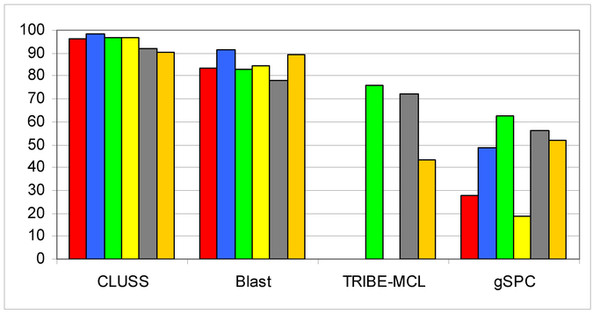
**Clustering results for the six subsets from COG**. For each algorithm (reading horizontally), the bars represent the *Q-measure *of the clustering results obtained on six randomly generated subsets: SS1, red; SS2, blue; SS3, green; SS4, yellow; SS5, gray; SS6, amber.

In the aim of comparing the efficiency of CLUSS to that of alignment-dependent clustering algorithms, we performed tests using CLUSS, BlastClust [7], TRIBE-MCL [8] and gSPC [9] on the COG database. In all performed comparisons, we used the default parameters of compared algorithms. We also used the widely known algorithm to compare protein sequences ClustalW [38] to calculate similarity matrices used by TRIBE-MCL [8] and gSPC [9]. Due to the complexity of alignment, these tests were done on six randomly generated subsets, named SS1 to SS6. The FASTA files of these subsets are provided as supplementary material [see Additional files 1, 2, 3, 4, 5 and 6]. The experimental results of each algorithm are summarized in Figure 4 for the obtained *Q-measures*, and Table 2 for the obtained numbers of clusters and the execution times. The detailed results using CLUSS are available as supplementary material [see Additional files 7, 8, 9, 10, 11 and 12]. BlastClust [7] yielded better results than TRIBE-MCL [8] and gSPC [9]. TRIB-MCL [8] obtained just one cluster for subsets SS1, SS2, SS4 and SS6. For each of the six subsets, the results show clearly that CLUSS obtained the best *Q-measure *compared to the other algorithms tested. Globally, the clusters obtained using our new algorithm CLUSS correspond better to the known characteristics of the biochemical activities and modular structures of the protein sequences. In Table 2 it can be seen that the fastest algorithm is BLAST, closely followed by our algorithm CLUSS, while TRIBE-MCL and gSPC, which use ClustalW [38] as similarity measures, are much slower than BLAST.

**Table 2:** Clustering results of the six subsets from the COG database. Number of clusters obtained by clustering the protein sequences of the six randomly generated subsets from the COG database (rows) with each of the clustering algorithms tested (columns). To each execution time of TRIBE-MCL [8] and gSPC [9], we added the corresponding execution time of ClustalW [38] used to compute the similarity matrix. Time is indicated in seconds.

Protein subsets	CLUSS		BLAST		MCL+ClustalW		SPC+ClustalW	
	
	Nbr	Time	Nbr	Time	Nbr	Time	Nbr	Time
SS1 (469 proteins)	30	106	114	14	1	495	9	499
SS2 (743 proteins)	15	234	102	58	1	1272	33	1275
SS3 (455 proteins)	30	114	132	18	8	586	27	588
SS4 (409 proteins)	19	82	125	11	1	452	4	454
SS5 (564 proteins)	35	103	172	15	6	538	30	540
SS6 (6444 proteins)	225	4272	732	583	1	95895	77	98880

### G-proteins

**Figure 5:**
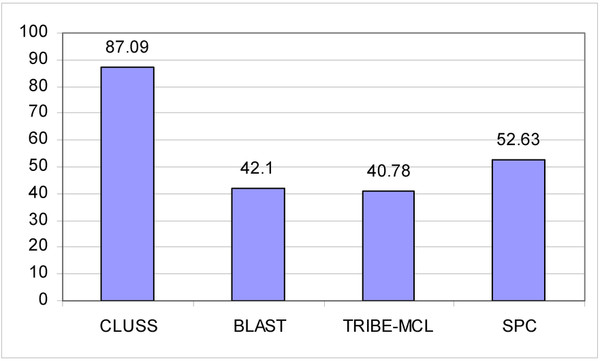
**Clustering results for the G-proteins**. For each algorithm (reading horizontally), the bars represent the *Q-measure *of the clustering results obtained on the members of the G-protein family. CLUSS obtained the highest quality measure of all the clustering results for this family, which shows that the CLUSS grouping is nearest to the functional reference classification for the G-protein family.

The G-proteins [32] (guanine nucleotide binding proteins) belong to the larger family of the GTPases. Their signalling mechanism consists in exchanging guanosine diphosphate (GDP) for guanosine triphosphate (GTP) as a general molecular function to regulate cell processes (reviewed extensively in [39]). This family has been the subject of a considerable number of publications by researchers around the world, so we considered it a good reference classification to test the performance of CLUSS. The sequences belonging to this family and the obtained clustering result are provided as supplementary material [see Additional files 13 and 14]. The experimental results obtained using the algorithms CLUSS, BlastClust [7], TRIBE-MCL [8] and gSPC [9], are summarized in Figure 5 for the obtained *Q-measures*, and Table 3 for the corresponding numbers of clusters and the execution time. The clustering results for the G-protein family show clearly that although this family is known to be easy to align, which should have facilitated the clustering task of the alignment-dependent algorithms, CLUSS yields a clustering with *Q-measure *value of **87.09%**, the highest of all the algorithms tested. Thus, the results obtained by CLUSS are much closer to the known classification of the G-protein family than those of the other tested algorithms are. In Table 3, we can make the same observation about the execution times of the different algorithms as in Table 2.

**Table 3:** Clustering results of the G-protein family. Number of clusters obtained by clustering the protein sequences of the G-protein family (rows) with each of the tested clustering algorithms (columns). Time is indicated in seconds. (The same remark applies as in Table 2 concerning TRIBE-MCL [8] and gSPC [9]).

Protein subsets	CLUSS		BLAST		MCL+ClustalW		SPC+ClustalW	
	
	Nbr	Time	Nbr	Time	Nbr	Time	Nbr	Time
G-proteins (381 proteins)	51	85	24	14	2	419	20	432

### Glycoside Hydrolase family 2 (GH2)

**Figure 6:**
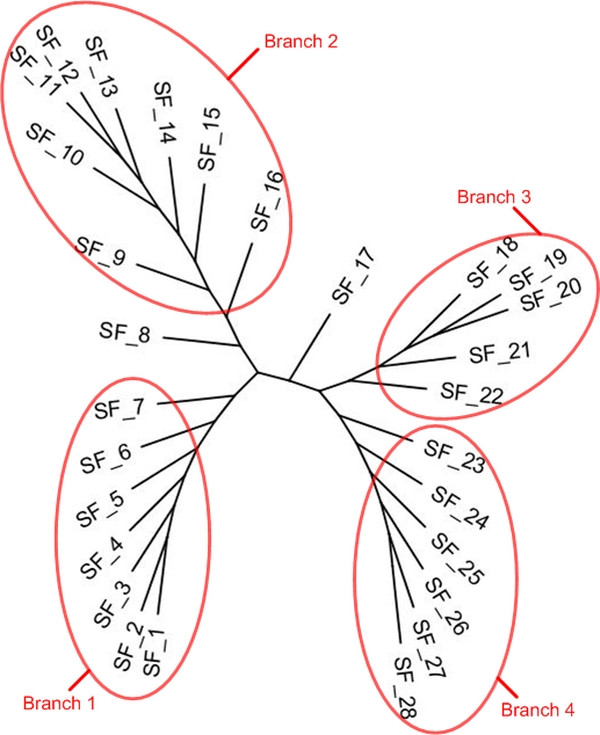
**CLUSS phylogenetic analysis of GH2 family**. The 316 enzymes of the GH2 family are clustered by CLUSS into 28 subfamilies (**SF_1 **to **SF_28**), in a phylogenetic tree composed of four main branches. Branches 1, 3 and 4 correspond to "*β-galactosidase*", "*β-mannosidase*" and "*β-glucuronidase*" activities, respectively. Most enzymes in branch 2 are labelled *as "putative β-galactosidases*" in databases. The "orphan" subfamily SF_17 includes nineteen sequences labelled as "*β*-galactosidases" in databases. Subfamily SF_8 contains "*exoglucosaminidase*" and "*endo-mannosidase*" activities.

To show the performances of CLUSS with multi-domain protein families which are known to be hard to align and have not yet been definitively aligned, experimental tests were performed on 316 proteins belonging to the Glycoside Hydrolases family 2 from the CAZy database (version of January 2006), the FASTA file is provided as supplementary material [see Additional file 15]. The CAZy database describes the families of structurally related catalytic and carbohydrate-binding modules or functional domains of enzymes that degrade, modify, or create glycosidic bonds. Among proteins included in CAZy database, the Glycoside Hydrolases are a widespread group of enzymes that hydrolyse the glycosidic bond between two or more carbohydrates or between a carbohydrate and a non-carbohydrate moiety. Among Glycoside Hydrolases families, the GH2 family, extensively studied at the biochemical level includes enzymes that perform five distinct hydrolytic reactions. Only complete protein sequences were retained for this study. In our experimentation, the GH2 proteins were subdivided into 28 subfamilies [see Additional file 16], organized in four main branches (see Figure 6). Three branches correspond perfectly to enzymes with known biochemical activities. The first branch (subfamilies 1–7) includes enzymes with "*β-galactosidase*" activity from both Prokaryotes and Eukaryotes. The third branch (subfamilies 18 to 22) groups enzymes with "*β-mannosidase*" activity, while the fourth branch (subfamilies 23 to 28) includes "*β-glucuronidases*".

The clustering scheme obtained warrants further comment. The "orphan" subfamily 17 includes nineteen sequences labelled as "*β-galactosidases*" in databases. While the branch 1 "*β-galactosidases*" are composed of five modules, known as the "*sugar binding domain*", the "*immunoglobulin-like β-sandwich*", the "*(αβ)8-barrel*", the "*β-gal small_N domain*" and the "*β-gal small_C domain*", the members of subfamily 17 lack the last two of these domains, which makes them more similar to "*β-mannosidases*" and "*β-glucuronidases*". These enzymes are distinct from those of branch 1 [40] and their separate localization is justified.

**Figure 7:**
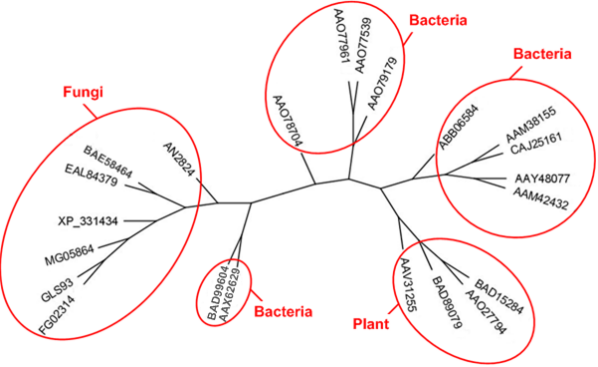
**Subfamily SF_8 phylogenetic analysis**. The phylogenetic tree of the 22 enzymes of subfamily SF_8 is grouped into (DDBJ: BAD89079, DDBJ: BAD15284) "*endo-β-mannosidasee*" and (GenBank: AAX62629, DDBJ: BAD99604) "*exo-β-D-glucosaminidase*" activities. Subfamily SF_8 also includes closely related plant enzymes and bacterial enzymes produced by members of the genus Xanthomonas, including several plant pathogens.

The second branch is the most heterogeneous in terms of enzyme activity. However, most of the subfamilies (9 to 16) group enzymes that are annotated as "*putative β-galactosidases*" in databases. To the best of our knowledge, none of these proteins, identified through genome sequencing projects, have been characterized by biochemical techniques, so their enzymatic activity remains hypothetical. At the beginning of this branch, subfamily 8 (shown in detail in Figure 7) groups enzymes characterized very recently: "*exo-β-glucosaminidases*" [41,42] and "*endo-β-mannosidases*" [43]. Again, theses enzymes share only three modules with the enzymes from branches 1, 3 and 4. The close proximity among "*exo-β-glucosaminidases*" and "*endo-β-mannosidases*" emerging from this work has not been described so far. Furthermore, subfamily 8 includes closely related plant enzymes with "*endo-β-mannosidase*" activity and bacterial enzymes produced by members of the genus *Xanthomonas*, including several plant pathogens. This could be an example of horizontal genetic transfer between members of these two taxa.

**Figure 8:**
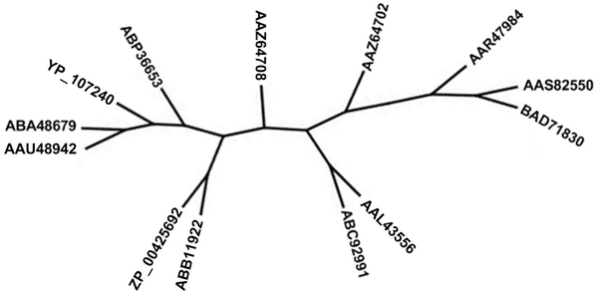
**Subfamily SF_22 phylogenetic analysis**. CLUSS has clustered in the same subfamily the enzymes GenBank: AAU48942 "*Burkholderia mallei*", **NCBI: YP_107240 **"*human*", GenBank: AAZ64708 "*Ralstonia eutropha*", GenBank: AAL43556 "*Agrobacterium tumefaciens*", GenBank: ABB11922 "*Burkholderia*" and **NCBI:ZP_00425692 **"*Burkholderia vietnamiensis*", which were recently analyzed by Côté *et al*. [41] and Fukamizo *et al*. [44] and characterized by their ability to recognize a substrate not yet associated with GH2 members.

Subfamily 22 (see Figure 8), also found at the beginning of a branch, has been recently analyzed by Côté *et al*. [41] and Fukamizo *et al*. [44], using structure-based sequence alignments and biochemical structure-function studies. It was shown that proteins from this subfamily have a different catalytic doublet and could recognize a new substrate not yet associated with GH2 members.

Globally, the clustering result for the GH2 proteins corresponds well to the known characteristics of their biochemical activities and modular structures. The results obtained with the CLUSS algorithm were highly comparable with those of the more complex analysis performed by Côté *et al*. [41] and Fukamizo *et al*. [44] using clustering based on structure-guided alignments, an approach which necessitates prior knowledge of at least one 3D protein structure.

### The 33 (*α*/*β*)8-barrel proteins from the GH2 family

The 33 (*α*/*β*)_8_-barrel proteins are a group within the GH2 family, studied recently by Côté *et al*. [41] and Fukamizo *et al*. [44]. The periodic character of the catalytic module known as "(*α*/*β*)_*8*_-*barrel*" makes these sequences hard-to-align using classical alignment approaches. The difficulties in aligning these modules are comparable to the problems encountered with the alignments of tandem-repeats, which have been exhaustively discussed [19]. The FASTA file and full clustering results of this subfamily are reported as supplementary material [see Additional files 17 and 18]. This group of 33 protein sequences includes "*β-galactosidase*", "*β-mannosidase*", "*β-glucuronidase*" and "*exo-β-D-glucosaminidase*" enzymatic activities, all of them extensively studied at the biochemical level. These sequences are multi-modular, with various types of modules, which complicate their alignment. Thus, the clustering of such protein sequences using the alignment-dependent algorithms becomes problematic. In our experiments, we tested quite a few known algorithms to align the 33 protein sequences, such as MUSCLE [45], ClustalW [38], MAFFT [46] and T-Coffee [47], etc. The alignment results of all these algorithms are in contradiction with those presented by Côté *et al*. [41], which in turn are supported by the structure-function studies of Fukamizo *et al*. [44]. This encouraged us to perform a clustering on this subfamily, to compare the behaviour of CLUSS with BlastClust [7], TRIBEMCL [8] and gSPC [9] to validate the use of CLUSS on the hard-to-align proteins. The experimental results with the different algorithms are summarized in Table 4, which shows the cluster correspondence of each of the sequences by algorithm used. An overview of the results is given below.

**Table 4:** Clustering results of the 33 (*α*/*β*)8-barrel protein sequences. The clustering correspondence of each of the 33 (*α*/*β*)_8_-barrel protein sequences (rows), obtained by Côté *et al*. [41] and Fukamizo *et al*. [44] and each of the clustering algorithms tested (columns). Each number in the table represents the corresponding cluster of the row's protein sequence obtained with the column's method. They are bold when they correspond to Côté *et al*. [41] and Fukamizo *et al*. [44] classification. The symbol "/" means that the row's protein sequence is unclustered.

Protein sequences	Côté Fukamizo	CLUSS	BLAST	MCL	SPC
GaEco	**1**	**1**	**1**	**1**	**1**
GaA	**1**	**1**	/	**1**	**1**
GaK	**1**	**1**	/	**1**	**1**
GaC	**1**	**1**	/	**1**	**1**
GaEcl	**1**	**1**	**1**	**1**	**1**
GaL	**1**	**1**	**1**	**1**	**1**
MaA	**2**	**2**	**2**	1	**2**
MaB	**2**	**2**	**2**	**2**	**2**
MaH	**2**	**2**	**2**	**2**	**2**
MaM	**2**	**2**	**2**	**2**	**2**
MaC	**2**	3	2	1	**2**
MaT	**2**	3	2	1	**2**
UnA	**3**	**3**	**3**	2	2
UnBv	**3**	**3**	**3**	2	2
UnBc	**3**	**3**	/	2	2
UnBm	**3**	**3**	**3**	2	2
UnBp	**3**	**3**	**3**	2	2
UnR	**3**	**3**	**3**	2	2
CsAo	**4**	**4**	/	1	**3**
CsS	**4**	**4**	**4**	1	**3**
CsG	**4**	**4**	**4**	1	**3**
CsM	**4**	**4**	**4**	1	**3**
CsN	**4**	**4**	/	1	**3**
CsAn	**4**	**4**	/	1	**3**
CsH	**4**	**4**	4	1	**3**
CsE	**4**	**4**	4	1	3
GIC	**5**	**5**	**5**	1	1
GIE	**5**	**5**	**5**	1	1
GIH	**5**	**5**	**5**	1	1
GIL	**5**	**5**	**5**	1	1
GIM	**5**	**5**	**5**	1	1
GIF	**5**	**5**	**5**	1	1
GIS	**5**	**5**	**5**	1	1

### CLUSS results

**Figure 9:**
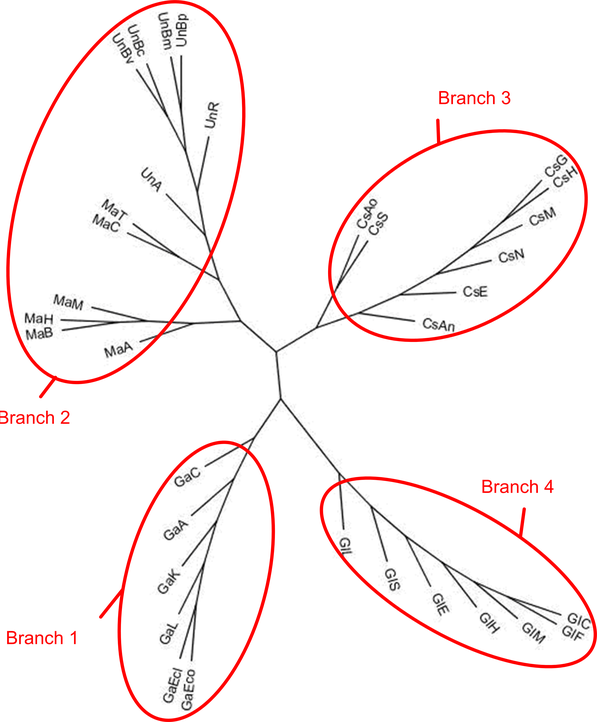
**33 (*α*/*β*)8-barrel group phylogenetic analysis**. The database entries of the 33 (*α*/*β*)_8_-barrel group are indicated: GaEco(GenBank: AAA24053), GaA(GenBank: AAA69907), GaK(GenBank: AAA35265), GaC(GenBank: AAA23216), GaEcl(DDBJ: BAA07673), GaL(GenBank: AAK06078), GIC(GenBank: AAC48809), GIE(GenBank: AAC74689), GIH(GenBank: AAA52561), GIL(GenBank: AAK07836), GIM(GenBank: AAA37696), GIF(GenBank: AAD01498), GIS(GenBank: AAR75615), MaA(EMBL: CAB63902), MaB(GenBank: AAC48460), MaC(GenBank: AAD42775), MaH(GenBank: AAC39573), MaM(GenBank: AAK18177), MaT(EMBL: CAD33708), CsAo(GenBank: AAX62629), CsS(DDBJ: BAC68933), CsG(NCBI: XM_382490), CsM(NCBI: XP_369600), CsN(NCBI: XP_331434), CsAn(GenBank: EAA63395), CsH(DDBJ: BAD99604), CsE(NCBI: XP_746417), UnA(GenBank: AAL43556), UnBv(GenBank: ABB11922), UnBc(NCBI: ZP_00425692), UnBm(GenBank: AAU48942), UnBp(NCBI: YP_107240), UnR(GenBank: AAZ64708).

The 33 (*α*/*β*)_8_-barrel proteins were subdivided by CLUSS into five subfamilies, organized in four main branches (see Table 5 and Figure 9). The first branch corresponds to the first cluster, which includes the enzymes with "*β-galactosidase*"activity; the second branch corresponds to the second and the third clusters, which include the enzymes with "*β-mannosidase*" activity; the third branch corresponds to the fourth cluster, which includes the enzymes with "*exo-β-D-glucosaminidase*" activity; and the fourth branch corresponds to the fifth cluster, which includes the enzymes with "*β-glucuronidase*" activity.

### BLAST results

The 33 (*α*/*β*)_8_-barrel proteins were subdivided into five subfamilies. Almost all the enzymes were clustered in the appropriate clusters, except for seven proteins that were unclustered, among which we find the following well-classified enzymes: "*β-galactosidase*" enzymes: GenBank: AAA69907, GenBank: AAA35265 and GenBank: AAA23216; "*β-mannosidase*" enzyme: NCBI:ZP_00425692; "*exo-β-D-glucosaminidase*" enzyme: GenBank: AAX62629.

### TRIBE-MCL results

The 33 (*α*/*β*)_8_-barrel proteins were subdivided by TRIBE-MCL into two mixed subfamilies. We find the "*β-mannosidase*" enzymes EMBL: CAB63902, GenBank: AAD42775 and EMBL: CAD33708 grouped in the "*β-galactosidase*"subfamily. Furthermore, the "*exo-β-D-glucosaminidase*" enzymes and the "*β-glucuronidases*" enzymes are grouped in the same subfamily.

### gSPC results

The 33 (*α*/*β*)_8_-barrel proteins were subdivided by gSPC into three subfamilies. Almost all the enzymes were grouped in the appropriate subfamily, except for the "*β-galactosidases*" and the "*β-glucuronidases*" which were grouped in the same subfamily.

Globally, the clustering of the 33 (*α*/*β*)_8_-barrel proteins generated by CLUSS corresponds better to the known characteristics of their biochemical activities and modular structures than do those yielded by the other algorithms tested. The results obtained with our new algorithm were highly comparable with those of the more complex, structure-based analysis performed by Côté *et al*. [41] and Fukamizo *et al*. [44].

### Other clustering tests

In our benchmarking (i.e., COG and G-proteins), we compared the execution times of SMS and ClustalW [38]; these results are provided as supplementary materials [see Additional file 19]. We also compared the performance of CLUSS with two other alignment-dependent algorithms, Secator [11] and COCO-CL [12]; the results again show the clear superiority of CLUSS. We also tested CLUSS on a variety of protein families and databases, such as the Clusters of Orthologous Groups for eukaryotic complete genomes database (KOG) [31], Glycoside Hydrolase family 8 (GH8) from the CAZy database [33] and the protein family known as the “Repressor, ORF, Kinases” (ROK) family [34]. Similarly to the results family shown in this section, all of these clusterings were highly concordant with their respective reference classifications. The FASTA files and the clustering results for the protein families and databases tested are available at the CLUSS website.

## Discussion

The alignment of protein sequences often provides information on conserved and mutated motifs, which is a good approach to measure the similarity between two protein sequences. The problem with this approach is that the result depends primarily on the alignability of the protein sequences, also on the algorithm selected and the parameters set by the user depending on the alignment algorithm used (e.g., gap penalties), which implies several different alignments with each algorithm. Such variations may create difficulties in measuring similarity between sequences and consequently complicate the clustering task. For the case of easy-to-align protein families, such as the G-protein family, almost all alignment algorithms find the same alignment for the conserved regions; however, the alignments of the less conserved regions are significantly different. On the other hand, for the case of hard-to-align protein families, such as the GH2 family, each alignment algorithm tends to diverge to its own, distinct results. Thus, in all cases, there is a significant need to develop efficient and robust alignment-independent approaches to clustering protein sequences.

The SMS developed in this paper makes it possible to measure the similarity between protein sequences based solely on the conserved motifs. The major advantage of SMS compared to the alignment-dependent approaches is that it gives significant results with protein sequences independent of their alignability, which allows SMS to be effective on both easy-to-align and hard-to-align protein families. This property is inherited by CLUSS, our new clustering algorithm, which uses SMS as its similarity measure. CLUSS used jointly with SMS is an effective clustering algorithm when used on protein sets with a restricted number of functions, which is the case of almost all protein families. It more accurately highlights the characteristics of the biochemical activities and modular structures of the clustered protein sequences than do the alignment-dependent algorithms.

So far, our similarity measure has been based on pre-determined substitution matrices. A possible future development is to propose an approach to automatically compute the weights of the conserved motifs instead of relying on pre-calculated substitution scores. There is also a need to speed up the extraction of the conserved motifs and the clustering of the phylogenetic tree, to scale the algorithm on datasets that are much larger in size with many more biological functions.

## Conclusion

Clustering of protein families into phylogenetically correct groups is a difficult problem, especially for those whose alignment is not biologically validated and not definitively performed. In this paper, we have proposed a new similarity measure, SMS, based on which we develop the new clustering algorithm CLUSS. CLUSS is applied directly to non-aligned sequences. Compared to existing clustering methods, CLUSS more accurately reflects the functional characteristics of the clustered families. It provides biologists with a new and plausible instrument for the analysis of protein sequences, especially those that cause problems for the alignment-dependent algorithms.

We believe that CLUSS can become an effective method and tool for clustering protein sequences to meet the needs of biologists in terms of phylogenetic analysis and function prediction. In fact, CLUSS gives an efficient evolutionary representation of the phylogenetic relationships between protein sequences. This algorithm constitutes a significant new tool for the study of protein families, the annotation of newly sequenced genomes and the prediction of protein functions, especially for proteins with multi-domain structures whose alignment is not definitively established. Finally, the tool can also be easily adapted to cluster other types of genomic data. The application server and the implementation are available at CLUSS website.

## Availability and requirements

Project name: CLUSS

Project home page: 

Operating system(s): MS Windows

Programming language: C++

Other requirements: /

License: Freely offered

Any restrictions to use by non-academics: /

## Abbreviations

GH2: Glycoside Hydrolase family 2

GH8: Glycoside Hydrolase family 8

COG: Clusters of Orthologous Groups of proteins

ROK: Repressor, ORF, Kinases

## Authors' contributions

AK designed, programmed and executed all experimentations with CLUSS and SMS, created the CLUSS web site, and wrote most of the manuscript. SW supervised the whole project, provided resources and wrote part of the manuscript. RB helped to design SMS and improve CLUSS through links with biological aspects, analyzed the results of clustering methods and wrote part of the manuscript. AF analyzed some results of the clustering method and helped in writing the manuscript. All authors read and approved the final manuscript.

## Supplementary Material

Additional file 1 Members of the SS1 subset from the COG family Click here for file

Additional file 2 Members of the SS2 subset from the COG family Click here for file

Additional file 3 Members of the SS3 subset from the COG family Click here for file

Additional file 4 Members of the SS4 subset from the COG family Click here for file

Additional file 5 Members of the SS5 subset from the COG family Click here for file

Additional file 6 Members of the SS6 subset from the COG family Click here for file

Additional file 7 Clustering result for the SS1 subset using CLUSS. Click here for file

Additional file 8 Clustering result for the SS2 subset using CLUSS Click here for file

Additional file 9 Clustering result for the SS3 subset using CLUSS. Click here for file

Additional file 10 Clustering result for the SS4 subset using CLUSS. Click here for file

Additional file 11 Clustering result for the SS5 subset using CLUSS. Click here for file

Additional file 12 Clustering result for the SS6 subset using CLUSS. Click here for file

Additional file 13 Members of the G-protein family Click here for file

Additional file 14 Clustering result of the G-Proteins family using CLUSS Click here for file

Additional file 15 Members of GH2 family Click here for file

Additional file 16 Clustering result for the GH2 family using CLUSS Click here for file

Additional file 17 Members of the 33 (*α*/*β*)_8_-barrel group from the GH2 family Click here for file

Additional file 18 Clustering result for the 33 (*α*/*β*)_8_-barrel group using CLUSS Click here for file

Additional file 19 Comparison between the execution times of SMS and ClustalW Click here for file
