# Supplementary material for: CLUSS: Clustering of protein sequences based on a new similarity measure
Source: BMC Bioinformatics. 2007 Aug 4;8:286. doi: 10.1186/1471-2105-8-286 (PMC1976428; doi:10.1186/1471-2105-8-286)
Supplement: Additional file 17 — Members of the 33 (α/β)8-barrel group from the GH2 family [file 1471-2105-8-286-S17.rtf]

>GaEco
MTMITDSLAVVLQRRDWENPGVTQLNRLAAHPPFASWRNSEEARTDRPSQQLRSLNGEWRFAWFPAPEAV
PESWLECDLPEADTVVVPSNWQMHGYDAPIYTNVTYPITVNPPFVPTENPTGCYSLTFNVDESWLQEGQT
RIIFDGVNSAFHLWCNGRWVGYGQDSRLPSEFDLSAFLRAGENRLAVMVLRWSDGSYLEDQDMWRMSGIF
RDVSLLHKPTTQISDFHVATRFNDDFSRAVLEAEVQMCGELRDYLRVTVSLWQGETQVASGTAPFGGEII
DERGGYADRVTLRLNVENPKLWSAEIPNLYRAVVELHTADGTLIEAEACDVGFREVRIENGLLLLNGKPL
LIRGVNRHEHHPLHGQVMDEQTMVQDILLMKQNNFNAVRCSHYPNHPLWYTLCDRYGLYVVDEANIETHG
MVPMNRLTDDPRWLPAMSERVTRMVQRDRNHPSVIIWSLGNESGHGANHDALYRWIKSVDPSRPVQYEGG
GADTTATDIICPMYARVDEDQPFPAVPKWSIKKWLSLPGETRPLILCEYAHAMGNSLGGFAKYWQAFRQY
PRLQGGFVWDWVDQSLIKYDENGNPWSAYGGDFGDTPNDRQFCMNGLVFADRTPHPALTEAKHQQQFFQF
RLSGQTIEVTSEYLFRHSDNELLHWMVALDGKPLASGEVPLDVAPQGKQLIELPELPQPESAGQLWLTVR
VVQPNATAWSEAGHISAWQQWRLAENLSVTLPAASHAIPHLTTSEMDFCIELGNKRWQFNRQSGFLSQMW
IGDKKQLLTPLRDQFTRAPLDNDIGVSEATRIDPNAWVERWKAAGHYQAEAALLQCTADTLADAVLITTA
HAWQHQGKTLFISRKTYRIDGSGQMAITVDVEVASDTPHPARIGLNCQLAQVAERVNWLGLGPQENYPDR
LTAACFDRWDLPLSDMYTPYVFPSENGLRCGTRELNYGPHQWRGDFQFNISRYSQQQLMETSHRHLLHAE
EGTWLNIDGFHMGIGGDDSWSPSVSAEFQLSAGRYHYQLVWCQK

>GaA
MSSSYITDQGPGSGLRVPARSWLNSDAPSLSLNGDWRFRLLPTAPGTPGAGSVLATGETVEAVASESFDD
SSWDTLAVPSHWVLAEDGKYGRPIYTNVQYPFPIDPPFVPDANPTGDYRRTFDVPDSWFESTTAALTLRF
DGVESRYKVWVNGVEIGVGSGSRLAQEFDVSEALRPGKNLLVVRVHQWSAASYLEDQDQWWLPGIFRDVK
LQARPVGGLTDVWLRTDWSGSGTITPEITADPAAFPVTLRVPELGLEVIWDSPADVAPVSIDAVEPWSAE
VPRLYDASVSSAAESISLRLGFRTVKIVGDQFLVNGRKVIFHGVNRHETNADRGRVFDEASAREDLALMK
RFNVNAIRTSHYPPHPRFLDLADELGFWVILECDLETHGFHALKWVGNPSDDPAWRDALVDRMERTVERD
KNHASIVMWSLGNESGTGANLAAMAAWTHARDLSRPVHYEGDYTGAYTDVYSRMYSSIPETDSIGRNDSH
ALLLGCNAIESARQRTRPFILCEYVHAMGNGPGAIDQYEDLVDKYPRLHGGFVWEWRDHGIRTRTADGTE
FFAYGGDFDEVIHDGNFVMDGMILSDSTPTPGLFEYKQIVSPIRLALTLNAEGNAGLTVANLRHTSDASD
VVLRWRVEHNGTRVDAGELTTDGANGPLQAGDSLTLTLPTIVAAAEGETWLSVEAVLREATAWAPAGHPL
SETQLDLSPAQPPLRVPRPASPIAGAAPVELGPATFDAGSLVTLAGLPVAGPRLELWRAPTDNDKGQGFG
AYGPEDPWINSGRGVPAPSSAVVWQQAGLDRLTRRVEDVAALPQGLRVRSRYAAANSEHDVAVEENWQLS
GDELWLRIDIAPSAGWDLVFPRIGVRLDLPSEVDGASWFGAGPRESYPDSLHSAVVGTHGGSLEELNVNY
ARPQETGHHSDVRWVELSRDGAPWLRIEADPDALGRRPGFSLAKNTAQEVALAPHPHELPESQHSYLYLD
AAQHGLGSRACGPDVWPDFALRPEARTLVLRIRAA

>GaK
MSCLIPENLRNPKKVHENRLPTRAYYYDQDIFESLNGPWAFALFDAPLDAPDAKNLDWETAKKWSTISVP
SHWELQEDWKYGKPIYTNVQYPIPIDIPNPPTVNPTGVYARTFELDSKSIESFEHRLRFEGVDNCYELYV
NGQYVGFNKGSRNGAEFDIQKYVSEGENLVVVKVFKWSDSTYIEDQDQWWLSGIYRDVSLLKLPKKAHIE
DVRVTTTFVDSQYQDAELSVKVDVQGSSYDHINFTLYEPEDGSKVYDASSLLNEENGNTTFSTKEFISFS
TKKNEETAFKINVKAPEHWTAENPTLYKYQLDLIGSDGSVIQSIKHHVGFRQVELKDGNITVNGKDILFR
GVNRHDHHPRFGRAVPLDFVVRDLILMKKFNINAVRNSHYPNHPKVYDLFDKLGFWVIDEADLETHGVQE
PFNRHTNLEAEYPDTKNKLYDVNAHYLSDNPEYEVAYLDRASQLVLRDVNHPSIIIWSLGNEACYGRNHK
AMYKLIKQLDPTRLVHYEGDLNALSADIFSFMYPTFEIMERWRKNHTDENGKFEKPLILCEYGHAMGNGP
GSLKEYQELFYKEKFYQGGFIWEWANHGIEFEDVSTADGKLHKAYAYGGDFKEEVHDGVFIMDGLCNSEH
NPTPGLVEYKKVIEPVHIKIAHGSVTITNKHDFITTDHLLFIDKDTGKTIDVPSLKPEESVTIPSDTTYV
VAVLKDDAGVLKAGHEIAWGQAELPLKVPDFVTETAEKAAKINDGKRYVSVESSGLHFILDKLLGKIESL
KVKGKEISSKFEGSSITFWRPPTNNDEPRDFKNWKKYNIDLMKQNIHGVSVEKGSNGSLAVVTVNSRISP
VVFYYGFETVQKYTIFANKINLNTSMKLTGEYQPPDFPRVGYEFWLGDSYESFEWLGRGPGESYPDKKES
QRFGLYDSKDVEEFVYDYPQENGNHTDTHFLNIKFEGAGKLSIFQKEKPFNFKISDEYGVDEAAHACDVK
RYGRHYLRLDHAIHGVGSEACGPAVLDQYRLKAQDFNFEFDLAFE

>GaC
MINNKPSLDWLENPEIFRVNRIDAHSDTWFYEKFEDVKLEDTMPLKQNLNGKWRFSYSENSSLRIKEFYK
DEFDVSWIDYIEVPGHIQLQGYDKCQYINTMYPWEGHDELRPPHISKTYNPVGSYVTFFEVKDELKNKQT
FISFQGVETAFYVWVNGEFVGYSEDTFTPSEFDITDYLREGENKLAVEVYKRSSASWIEDQDFWRFSGIF
RDVYLYAVPETHVNDIFIKTDLYDDFKNAKLNAELKMIGNSETTVETYLEDKEGNKIAISEKIPFSDELT
LYLDAQNINLWSAEEPNLYTLYILVNKKDGNLIEVVTQKIGFRHFEMKDKIMCLKWKRIIFKGVNRHEFS
ARRGRSITKEDMLWDIKFLKQHNINAVRTSHYPNQSLWYRLCDEYGIYLIDETNLESHGSWQKMGQIEPS
WNVPGSLPQWQAAVLDRASSMVERDKNHPSVLIWSCGNESYAGEDIYQMSKYFRKKDPSRLVHYEGVTRC
REFMTRRHESRMYAKAAEIEEYLNDNPKKPYISCEYMHSMGNSTGGMMKYTELEDKYLMYQGGFIWDYGD
QALYRKLPDGKEVLAYGGDFTDRPTDYNFSGNGLIYADRTISPKAQEVKYLYQNVKLEPDEKGVTIKNQN
LFVNTDKYDLYYIVERDGKLIKDGYLNVSVAPDEEKYIELPIGNYNFPEEIVLTTSLRLAQATLWAEKGY
EIAFGQKVIKEKSDMNNHNSESKMKIIHGDVNIGVHGKDFKAIFSKQEGGIVSLRYNNKEFITRTPKTFY
WRATTDNDRGNRHEFRCSQWLAATMGQKYVDFSVEEFDEKITLYYTYQLPTVPSTNVKITYEVSGEGIIK
VNVKYKGVSGLPELPVLGMDFKLLAEFNSFSWYGMGPEENYIDRCEGAKLGIYESTQ

>GaEcl
MSDTPSLTLSALLARRDWENPVVTQWNRLAAHAPLHSWRNEPSARDDAGSARRQTLNGLWRFSYFTAPEQ
VPQAWVTEDCADAVAMPVPSNWQMQGFDTPIYTNVTYPIPVNPPFVPQENPTGCYSLTFEVDDAWLQSGQ
TRIIFDGVNSAFHLWCNGQWIGYSQDSRLPAEFDLSAALRPGQNRLAVMVLRWCDGSYLEDQDMWRMSGI
FRDVTLLHKPETQIADYHVVTDLNAELDRAVLKVDVTLAGAGFADGEVVFTLWRKGEKCASVSRQPGSAI
VDERGSWAERLTVTIPVETPALWSAETPELYRLTMALLNPQGEVLEIEACDVGFRRVEISNGLLKLNGKP
LLIRGVNRHEHHSENGQVMDEATMRRDIETMKQHSFNAVRCSHYPNHPLWYQLCDRYGLYVVDEANIETH
GMVPMSRLADDPRWLPAMSERVTRMVQRDRNHPSIIIWSLGNESGHGANHDALYRWLKTTDPTRPVQYEG
GGANTAATDIVCPMYARVDRDQPFPAVPKWSIKKWIGMPDETRPLILCEYAHAMGNSFGGFAKYWQAFRS
HPRLQGGFVWDWVDQALTKRDEDGNTFWAYGGDFGDKPNDRQFCLNGLVFPDRTPHPALYEAHGPQQFFT
FTRVSTSPLVIEVQSGYLFRHTDNEVLNWTVARDGDVLVAGEVTLAMVPEGTQRLEIALPELNAGPGEVW
LNVEVRQPRATPWSPAAIAAAGSSGRFRLRSLLLRQPRRRAAVLTQTDRILEIAHRQQRWQFDRASGNLT
QWWRNGVETLLSPLTDNVSRAPLDNDIGVSEATKIDPNAWVERWKAAGMYDLTPRVLHCEAEQHAGEVVV
TTQHVLEYRGKALFLSRKVWRIDEQGVLHGDIQVDMASDIPEPARIGLSVHLAETPENVRWLGLGPHENY
PDRKLAAQQGRWTLPLEAMHTPYIFPTENGLRCDTRELVVGMHQLNGHFHFSVSRYSQQQLRETTHHHLL
REEPGCWLNLDAFHMGVGGDDSWSPSVSPEFILQTRQLRYTFSWQQNP

>GaL
MMTMIDVLERKDWENPVVSNWNRLPMHTPMDLLEKQSLNGLWNFDHFSRISDVPKNWLELTESKTEIIVP
SNWQIEFKDKSDVPIYTNVTYPIPIQPPYVPEANPVGAYSRYFDITKEWLESGHVHLTFEGVGSAFHFWL
NGEYGGYSEDSRLPAEFDISNLAKEGQNCLKVLVFRWSKVTYFEDQDMWRMSGIFRSVNLQWLPDNYLLD
FSIKTDLDEDLDFANVKLQAYAKNIDDACLEFKLYDDEQLIGECHGFDAEIGVVNPKLWSDEIPYLYRLE
LTLMDRSGAVFHKETKKIGIRKIAIEKGQLKINGKALLVRGVNKHEFTPEHGYVVSEEVMIKDIKLMKEH
NFNAVRCSHYPNDSRWYELCDEYGLYVMDEANIETHGMTPMNRLTNDPTYLPLMSERVTRMVMRDRNHPS
IIIWSLGNESGYGSNHQALYDWCKSFDSSRPVHYEGGDDASRGATDATDIICPMYARVDSPSINAPYSLK
TWMGVAGENRPLILCEYAHDMGNSLGGFGKYWQAFREIDRLQGGFIWDWVDQGLLKDGNYAYGGDFGDKP
NDRQFSLNGLVFPNRQAKPALREAKYWQQYYQFELEKTPLGQVFAFTVTNEYLFRSTDNEKLCYQLINGL
EVLWENELILNMPAGGSMRIDLSELPIDGTDNLFLNIQVKTIEKCNLLESDFEVAHQQFVLQEKINFTDR
IDSNEEITLFEDEELLTVRSAKQKFIFNKSNGNLSRWLDEKGNEKLLHELSEQFTRAPLDNDIGVSEVEH
IDPNAWLERWKGIGFYELKTLLKTMIIQATENEVIISVQTDYEAKGKIAFSTIREYHIFRNGELLLKVDF
KRNIEFPEPARIGLSLQLAEKAENVTYFGLGPDENYPDRRGASLFGQWNLRITDMTTPYIFPSENGLRME
TRELNYDRLKVRAMGQSFAFNLSPYSQNQLAKKGHWHLLEEEAGTWLNIDGFHMGVGGDDSWSPSVAQEY
LLTKGNYHYEVSFKLT

>GIC
MSRGPAGAWVALGPLLWTCGLALEGGMLYPRESPSRERKDLDGLWSFRADFSDGRRQGFEQQWYRAPLRE
SGPTLDMPVPSSFNDVGQDRQLRSFVGWVWYEREATLPRRWSQDPGTRVVLRIGSAHYYAIVWVNGVHVA
EHEGGHLPFEADISKLVQSGPLSSCRITLAINNTLTPHTLPPGTIVYKTDASKYPKGYFVQNTYFDFFNY
AGLHRPVLLYTTPTTYIDDITVTTGVDQDTGLVDYQIFVQGSEHFQLEVYLLDEEGKVVAQGTGSQGRLQ
VPNVHLWWPYLMHEHPAYLYSLEVRLTAQMAAGPVSDFYTLPVGIRTVAVTERQFLINGKPFYFHGVNKH
EDADIRGKGFDWPLLVKDFNLLRWLGANAFRTSHYPYAEEVMQLCDRYGIVVIDESPGVGIMLVQSYSNV
SLQHHLEVMGELVRRDKNHPSVVMWSVANEPTSFLKPAAYYFKTLIAHTKALDPSRPVTFVTNSNYEADL
GAPYVDVICVNSYYSWYHDYGHMEVIQLQLATEFENWYRTYQKPIIQSEYGAETIAGFHQDPPLMFSEEY
QKGLLEQYHLVLDQKRKEYVVGELIWNFADFMTDQSPQRAVGNRKGIFTRQRQPKAAAFLLRERYWKLAN
ETGHHRSAAKSQCLENSPFAL

>GIE
MLRPVETPTREIKKLDGLWAFSLDRENCGIDQRWWESALQESRAIAVPGSFNDQFADADIRNYAGNVWYQ
REVFIPKGWAGQRIVLRFDAVTHYGKVWVNNQEVMEHQGGYTPFEADVTPYVIAGKSVRITVCVNNELNW
QTIPPGMVITDENGKKKQSYFHDFFNYAGIHRSVMLYTTPNTWVDDITVVTHVAQDCNHASVDWQVVANG
DVSVELRDADQQVVATGQGTSGTLQVVNPHLWQPGEGYLYELCVTAKSQTECDIYPLRVGIRSVAVKGEQ
FLINHKPFYFTGFGRHEDADLRGKGFDNVLMVHDHALMDWIGANSYRTSHYPYAEEMLDWADEHGIVVID
ETAAVGFNLSLGIGFEAGNKPKELYSEEAVNGETQQAHLQAIKELIARDKNHPSVVMWSIANEPDTRPQG
AREYFAPLAEATRKLDPTRPITCVNVMFCDAHTDTISDLFDVLCLNRYYGWYVQSGDLETAEKVLEKELL
AWQEKLHQPIIITEYGVDTLAGLHSMYTDMWSEEYQCAWLDMYHRVFDRVSAVVGEQVWNFADFATSQGI
LRVGGNKKGIFTRDRKPKSAAFLLQKRWTGMNFGEKPQQGGKQ

>GIH
MARGSAVAWAALGPLLWGCALGLQGGMLYPQESPSRECKELDGLWSFRADFSDNRRRGFEEQWYRRPLWE
SGPTVDMPVPSSFNDISQDWRLRHFVGWVWYEREVILPERWTQDLRTRVVLRIGSAHSYAIVWVNGVDTL
EHEGGYLPFEADISNLVQVGPLPSRLRITIAINNTLTPTTLPPGTIQYLTDTSKYPKGYFVQNTYFDFFN
YAGLQRSVLLYTTPTTYIDDITVTTSVEQDSGLVNYQISVKGSNLFKLEVRLLDAENKVVANGTGTQGQL
KVPGVSLWWPYLMHERPAYLYSLEVQLTAQTSLGPVSDFYTLPVGIRTVAVTKSQFLINGKPFYFHGVNK
HEDADIRGKGFDWPLLVKDFNLLRWLGANAFRTSHYPYAEEVMQMCDRYGIVVIDECPGVGLALPQFFNN
VSLHHHMQVMEEVVRRDKNHPAVVMWSVANEPASHLESAGYYLKMVIAHTKSLDPSRPVTFVSNSNYAAD
KGAPYVDVICLNSYYSWYHDYGHLELIQLQLATQFENWYKKYQKPIIQSEYGAETIAGFHQDPPLMFTEE
YQKSLLEQYHLGLDQKRRKYVVGELIWNFADFMTEQSPTRVLGNKKGIFTRQRQPKSAAFLLRERYWKIA
NETRYPHSVAKSQCLENSPFT

>GIL
MESALYPIQNKYRFNTLMNGTWQFETDPNSVGLDEGWNKELPDPEEMPVPGTFAELTTKRDRKYYTGDFW
YQKDFFIPSFLKKKELYIRFGSVTHRAKVFINGHEVGQHEGGFLPFQVKISNYINYDQTNRVTVLVNNEL
SEKAIPCGTEEILDNGQKLAQPYFDFFNYSGIMRNVWLLALPQSQITNFKLNYQLANNKATITYNIEANN
NAEFKVTLFDNQKEVACATSKNTSSLTIKNPHLWSPNDPYSYKIKIEMLEDGKTVDEYTDKIGIRTVKIV
NDKILLNNHPIYLKGFGKHEDFNVLGKAVNESIIKRDYECMKWIGANCFRSSHYPYAEEWYQYADKYGFL
IIDEVPAVGLNRSITNFLNVTNSNQSHFFASKTVPELKKVHEQEIKEMIDRDQRHPSVIAWSLFNEPEST
TQESYDYFKDIFAFARKLDPQNRPYTGTLVMGSGPKVDKLHPLCDFVCLNRYYGWYVAGGPEIVNAKKML
EDELDGWQNLKLNKPFVFTEFGADTLSSSHRLPDEMWSQEYQNEYYQMYFDIFKKYPFICGELVWNFADF
KTSEGIMRVGGNDKGIFTRDREPKDIAFTLKKRWQQLN

>GIM
MSLKWSACWVALGQLLCSCALALKGGMLFPKESPSRELKALDGLWHFRADLSNNRLQGFEQQWYRQPLRE
SGPVLDMPVPSSFNDITQEAALRDFIGWVWYEREAILPRRWTQDTDMRVVLRINSAHYYAVVWVNGIHVV
EHEGGHLPFEADISKLVQSGPLTTCRITIAINNTLTPHTLPPGTIVYKTDTSMYPKGYFVQDTSFDFFNY
AGLHRSVVLYTTPTTYIDDITVITNVEQDIGLVTYWISVQGSEHFQLEVQLLDEDGKVVAHGTGNQGQLQ
VPSANLWWPYLMHEHPAYMYSLEVKVTTTESVTDYYTLPVGIRTVAVTKSKFLINGKPFYFQGVNKHEDS
DIRGKGFDWPLLVKDFNLLRWLGANSFRTSHYPYSEEVLQLCDRYGIVVIDECPGVGIVLPQSFGNESLR
HHLEVMEELVRRDKNHPAVVMWSVANEPSSALKPAAYYFKTLITHTKALDLTRPVTFVSNAKYDADLGAP
YVDVICVNSYFSWYHDYGHLEVIQPQLNSQFENWYKTHQKPIIQSEYGADAIPGIHEDPPRMFSEEYQKA
VLENYHSVLDQKRKEYVVGELIWNFADFMTNQSPLRVIGNKKGIFTRQRQPKTSAFILRERYWRIANETG
GHGSGPRTQCFGSRPFTF

>GIF
MLRGPAAVWAALGPLLWACGLALRGGMLYPRESPSRERKELNGLWSFRADFSENRRQGFEQQWYRTPLRE
SGPTLDMPVPSSFNDVGQDRQLRSFVGWVWYEREATLPQRWTQDLGTRVVLRIGSAHYYAIVWVNGVHVA
EHEGGHLPFEADISKLVQSGPLASCRITIAINNTLTPHTLPPGTILYQTDTSKYPKGYFVQNINFDFFNY
AGLHRPVLLYTTPTTYIDDITISTSVNQDTGLVDYQIFVEGGEHFQLEVRLLDEEGKVVAQGTGGRGQLQ
VPNAHLWWPYLMHEHPAYLYSLEVRLTAQTAAGSVSDFYTLPVGIRTVAVTEHQFLINGKPFYFHGVNKH
EDADIRGKGFDWPLLVKDFNLLRWLGANAFRTSHYPYAEEVMQLCDRYGIVVIDESPGVGIVLVESYSNV
SLQHHLEVMEELVRRDKNHPAVVMWSVANEPASFLKPAGYYFKTLIAHTKALDPSRPVTFVTNSNYEADL
GAPYVDVICVNSYYSWYHDYGHMEVIQLQLATQFENWYRTYQKPIIQSEYGADTIAGFHQDPPLMFSEEY
QKGLLEQYHLVLDQKRKEYVVGELIWNFADFMTNQSPQRVMGNKKGIFTRQRQPKGAAFLLRERYWKLAN
ETRYPWSAVKSQCLENSPFTL

>GIS
MLYPINTETRGVFDLNGVWNFKLDYGKGLEEKWYESKLTDTISMAVPSSYNDIGVTKEIRNHIGYVWYER
EFTVPAYLKDQRIVLRFGSATHKAIVYVNGELVVEHKGGFLPFEAEINNSLRDGMNRVTVAVDNILDDST
LPVGLYSERHEEGLGKVIRNKPNFDFFNYAGLHRPVKIYTTPFTYVEDISVVTDFNGPTGTVTYTVDFQG
KAETVKVSVVDEEGKVVASTEGLSGNVEIPNVILWEPLNTYLYQIKVELVNDGLTIDVYEEPFGVRTVEV
NDGKFLINNKPFYFKGFGKHEDTPINGRGFNEASNVMDFNILKWIGANSFRTAHYPYSEELMRLADREGL
VVIDETPAVGVHLNFMATTGLGEGSERVSTWEKIRTFEHHQDVLRELVSRDKNHPSVVMWSIANEAATEE
EGAYEYFKPLVELTKELDPQKRPVTIVLFVMATPETDKVAELIDVIALNRYNGWYFDGGDLEAAKVHLRQ
EFHAWNKRCPGKPIMITEYGADTVAGFHDIDPVMFTEEYQVEYYQANHVVFDEFENFVGEQAWNFADFAT
SQGVMRVQGNKKGVFTRDRKPKLAAHVFRERWTNIPDFGYKN

>MaA
MRHSIGLAAALLAPTLPVALGQHIRDLSSEKWTLSSRALNRTVPAQFPSQVHLDLLRAGVIGEYHGLNDF
NLRWIAAANWTYTSQPIKGLLDNYGSTWLVFDGLDTFATISILWTANRIHGQSVSPVSGSMYLPALEACQ
RRILIRKVSFRGGVTAEVNTCYLHIEWPDDVQLTYEYPNRWFMRKEQSDFGWDWGPAFAPAGPWKPAYIV
QLDKKESVYVLNTDLDIYRKNQINYLPPDQSQPWVVNASIDILGPLPAKPTMSIEVRDTHSGTILTSRTL
NNVSVAGNAITGVTVLDGLNPKLWWPQSSVIRTSTMFLSLSKVEGTRPWPVWTNGRASAPFFLNQRNITE
VQRAQGIAPGANWHFEVNGHEFYAKGSNLIPPDSFWTRVTEERISRLFDAVVVGNQNMLRVWSSGAYLHD
YIYDLADEKGILLWSEFEFSDALYPSDDAFLENVAAEIVYNVRRVNHHPSLALWAGGNEIESLMLPRVKD
AAPSSYSYYVGEYEKMYISLFLPLVYENTRSISYSPSSTTEGYLYIDLSAPVPMAERYDNTTSGSYYGDT
DHYDYDTSVAFDYGSYPVGRFANEFGFHSMPSLQTWQQAVDTEDLYFNSSVVMLRNHHDPAGGLMTDNYA
NSATGMGEMTMGVISYYPIPSKSDHISNFSAWCHATQLFQADMYKSQIQFYRRGSGMPERQLGSLYWQLE
DIWQAPSWAGIEYGGRWKVLHHVMRDIYQPVIVSPFWNYTTGSLDVYVTSDLWSPAAGTVDLTWLDLSGR
PIAGNAGTPKSVPFTVGGLNSTRIYGTNVSSLGLPDTKDAVLILSLSAHGRLPNSDRTTNLTHENYATLS
WPKDLKIVDPGLKLGYSSKKTTVTVEATSGVSLYTWLDYPEGVVGYFEENAFVLAPGEKKEIGFTVLDDT
TNGAWVRNITVQSLWDQKVRG

>MaB
MLLRLLLLLAPCGAGFATKVVSISLRGNWKIHSGNGSLQLPATVPGCVHSALFNKRIIKDPYYRFNNLDY
RWIALDNWTYIKKFKLHSDMSTWSKVNLVFEGIDTVAVVLLNSVPIGKTDNMFRRYSFDITHTVKAVNII
EVRFQSPVVYANQRSERHTAYWVPPNCPPPVQDGECHVNFIRKMQCSFGWDWGPSFPTQGIWKDVRIEAY
NVCHLNYFMFTPIYDNYMKTWNLKIESSFDVVSSKLVSGEAIVAIPELNIQQTNNIELQHGERTVELFVK
IDKAIIVETWWPHGHGNQTGYNMSVIFELDGGLRFEKSAKVYFRTVELVEEPIQNSPGLSFYFKINGLPI
FLKGSNWIPADSFQDRVTSAMLRLLLQSVVDANMNALRVWGGGVYEQDEFYELCDELGIMIWQDFMFACA
LYPTDKDFMDSVREEVTHQVRRLKSHPSIITWSGNNENEAALMMGWYDTKPGYLQTYIKDYVTLYVKNIR
TIVLEGDQTRPFITSSPTNGAKTIAEGWLSPNPYDLNYGDVHFYDYVSDCWNWRTFPKARFVSEYGYQSW
PSFSTLEKVSSEEDWSYRSSFALHRQHLINGNNEMLHQIELHFKLPNSTDQLRRFKDTLYLTQVMQAQCV
KTETEFYRRSRSEIVNGKGHTMGALYWQLNDIWQAPSWSSLEYGGKWKMLHYFARHFFAPLLPVGFEDKD
MLFIYGASHLHSDQQMMLTVRVHTWSSLELVCSESTNPFVIKAGESVLLYTKPVPELLKGCPGCTRQSCV
VSFYLSTDGELLSPINYHFLSSLKNAKGLHKANITATISQQGDTFVFDLKTSAVAPFVWLDVGSIPGRFS
DNGFLMTEKTRTVFFYPWKPTSKSELEQSFHVTSLADTY

>MaC
MITQDLYDGWTLTAVSGPVPAELAGVRVRARVPGTSHTALLDEGLIPDPYLDRNEDVLAWMRRTDWAYER
ELVLDPAAADERVDLVFGGIDTVGTVTFDGHELGRTANQHRSYRFDVRALLRPDTQRLRVDLRAAIVHAE
AERERLGHRPLAYPQPFNMVRKMACSFGWDWGPDLQTAGLWKPVRVERWRTARLASVRTHVTVDPDGTGR
VRVLVDLERSGLPGGDAPVTLRARVLSADVAVTVPGTATSAVVELEVPRAPLWWPVGHGPQPLSDLTVTL
ATRDDEPLDSWSRRIGFRTVEVDTTPDEDGTPFTFRVNGRPVFVKGANWIPDDHLLTRITRERLAHRLDQ
AVEANLNLLRVWGGGIYESEDFYDLCDERGLLVWQDFLLACAAYPEEQPIWDELEAEARENVARLTPHAS
LVLWNGGNENLWGFMDWGWPQELEGRTWGYRLATELLKGVVAELDPTRPYADGSPYSPGFALDDVHPNDP
DHGTHHEWEVWNRVDYSAYRDDVPRFCSEFGFQGPPTWSTLTRAVRADDGGPLTKDDPTFLLHQKAEDGN
GKLDRGLAPHLGVPAGFVDWHWATQLNQARAVAFAIEHYRSWWPRTAGAIVWQLNDCWPVTSWAAIDGDE
RVKPLWHALRRAYAPRLLTVQPRDGRDELAVVNDTGGLWQGTVRLSRRTLDGATLAEVELGLAVGAWSVG
LFALPDEVAAPDDAAGEVLVVDLGDVRTVHTWAQDVDLRLDPDPVSATVSPLQDGYRVDVTARTFARSVT
LHVDRLDPDATVDDALVDVPAGETFSFHVRTSARFDAAALTRSPVLRTANDVVVPRGATAPAGAERDLQQ
SR

>MaH
MRLHLLLLLALCGAGTTAAELSYSLRGNWSICNGNGSLELPGAVPGCVHSALFQQGLIQDSYYRFNDLNH
RWVSLDNWTYSKEFKIPFEISKWQKVNLILEGVDTVSKILFNEVTIGETDNMFNRYSFDITNVVRDVNSI
ELRFQSAVLYAAQQSKAHTXYQVPPDCPPLVQKGECHVNFVRKEQCSFSWDWGPSFPTQGIWKDVRIEAY
NICHLNYFTFSPIYDKSAQEWNLEIESTFDVVSSKPVGGQVIXAIPKLQTQQTYSIELQPGKRIVELFVN
ISKNITVETWWPHGHGNQTGYNMTVLFELDGGLNIEKSAKVYFRTVELIEEPIKGSPGLSFYFKINGFPI
FLKGSNWIPADSFQDRVTSELLRLLLQSVVDANMNTLRVWGGGIYEQDEFYELCDELGIMVWQDFMFACA
LYPTDQGFLDSVTAEVAYQIKRLKSHPSIIIWSGNNENEEALMMNWYHISFTDRPIYIKDYVTLYVKNIR
ELVLAGDKSRPFITSSPTNGAETVAEAWVSQNPNSNYFGDVHFYDYISDCWNWKVFPKARFASEYGYQSW
PSFSTLEKVSSTEDWSFNSKFSLHRQHHEGGNKQMLYQAGLHFKLPQSTDPLRTFKDTIYLTQVMQAQCV
KTETEFYRRSRSEIVDQQGHTMGALYWQLNDIWQAPSWASLEYGGKWKMLHYFAQNFFAPLLPVGFENEN
TFYIYGVSDLHSDYSMTLSVRVHTWSSLEPVCSRVTERFVMKGGEAVCLYEEPVSELLRRCGNCTRESCV
VSFYLSADHELLSPTNYHFLSSPKEAVGLCKAQITAIISQQGDIFVFDLETSAVAPFVWLDVGSIPGRFS
DNGFLMTEKTRTILFYPWEPTSKNELEQSFHVTSLTDIY

>MaM
MHLHLLLILALFRAGCVVAGPSYSLSGSWRVSNGNGSLELPATVPGYVHSALHQHGLIQDPYYRFNDLNY
RWISLDNWTYSTEFKIPFNLSEWQKVKLIFDGVDTVAEILFNNVTIGKTDNMFTGYSFDVTNVVKDVNSL
KLQFRSAVQYAECQSKAHTSYRVPPECPPVEQKGECHVNFIRKAQCSFSWDWGPSFPSQGIWKDVRIEAY
NIAHLDYLTFLPVYDNASQAWNIEIEASFDVASSKSVSGQVTVAIPQLKTQQTNDIELQQEQRIVKLLVK
IRKDVAVETWWPRGHGNQTGYNMTILFALDGGLKIEKAAKVYFRTVQLIEEGIKGSPGLSFYFKINGLPI
FLKGSNWIPADSFQDKVTSDRLQLLFQSVVDANMNTLRVWGGGIYEQDEFYALCDELGIMVWQDFMFASA
LYPTEPGFLASVRKEVTYQVRRLKSHPSIIIWSGNNENEVALSVNWFHVNPRDMKTYIDDYVTLYVKNIR
KIVLSEDKSRPFIASSPTNGMKTMEEGWISYDPYSIQYGDIHFYNYADDCWNWKIFPKARLVSEYGYQSW
PSFSTLEKVSSQEDWAYNSRFSLHRQHHEDGNHQMLHQVKMHFKLPQGTDPLRTFKDTIYLTQVMQAQCI
KTETEFYLRSRSEIVDGKGHTMGALYWQLNDIWQAPSWASLEYGGKWKMLHYFARRFFAPLLPVGFEDEG
VFYVYGVSDLHKDHHTQLTVRLHHWSSPKPLCSLVNSSIVVKAGEAVVLFQMPVSELLKRCRGCTRETCV
VSFYFSTDKELFSPTNYHFLSSLKDAKGLLEANITVNISQKGNVFVFDLETSAVAPFVWLDVGSIPGRFS
DNGFLMIRKKLSVLFYPWKPTSKSELQQAFSVTSLTDTY

>MaT
MQHRFTCGDAMAVRIELRENWVLRADDPAEVPVEIGPAGIPATVPGCVHTDLMAANLIPDPYQGRNETEL
GWIGRTQWSYTTTFDATALAEAERIDLECAGLDTVATVFLNGTEVGQSRNMHRSYRFDLRRALRDGTNEL
RVEFASPYSYATALRDKLGDRPNAYPEPFQFIRKMACNFGWDWGPTLVTSGIWRPIHVHAWHTARLAQVV
PLITVARTHDGSLEGRVLVRVEVERSEHGEDTELVVRVRIADREQSIAVPAGVCRTELEVTVPDPDLWWP
RGYGDQPLYDLRVDLAAHGEELDTWQRRIGFRTVELDTGVDEDGRRFTIVVNGVPVLVKGANWIPDDCFV
SRVGRDRYAARIDQAVAANMNLLRVWGGGRYESEDFYELCDERGILVWQDFLFACAAYPEEPPITEEVEA
EAREVVARLAPYPSLVLWNGNNENIWGYWDWGWKEELAGRSWGEGYYLELLPRIVAEVDPTRPYWPGSPY
SGVPDIHPNDPRYASIHIWDVWNEVDYTAYRNYRPRFVAEFGFQAPPTYATLRSALPGEELRPDSPGMLH
HQKAVDGNGKLARGLAPHFGNPADFDDWHYLTQVNQARAITLGIEHFRAQWPRCTGSVVWQLNDCWPVTS
WSAVDGEGRRKPLWYALRAVYAERLATVQPDGDGLVLVVANDSNREWTGTAHLARRALDGTVLAAADAPF
TVPARGAVRVPLPAEVARPGDATRELITADTGARRAYWFFAEDREIAYPAPEYDTRVVPRGADLCVTVTA
RSLLRDLSLFADRLDPGAEADDMLVTLLPGESHTFTVRGGAHLDPAQVVQPPVLRTVNDALRAAQDSIPV

>CsAo
MSFRQKRTRIPLLAMTVTALAAAVCGVTTAPAATGAEVAVPLSVGAAAGNATPIPGYVIQSSAQVSDDSA
VSKPGFPTSGWYPVSSRSTVYAGLLQNGKYADPFYSTNMQNVPAAQFSVPWWYRTDLNVDDTSSRTYLDF
SGVLSKADVWVNGTKVATKDQVNGAYTRHDLDITAQVHTGVNSVAFKVYPNDPNRDLSMGWIDWAQTPPD
QNMGIVRDVLVRRSGAVALRSAHVIQKLNSALDHADLTVKADVRNDSANAVQTTVAGTVAGKPISQTVSL
AAKERKTVTFPLVGLDRPNVWWPAGMGGQHRYDLDLTASVGGTPSDAAKSKFGVRDVKATLNSSGGRQYS
VNGKPLLIRGGGYTPDLFLRWNETAAADKLKYVLNLGLNTVRLEGHIEPDEFFDIADDLGVLTMPGWECC
DKWEGQVNGEEKGEPWVESDYPIAKASMFSEAERLRDHPSVISFHIGSDFAPDRRIEQGYLDAMKAADFL
LPVIPAASARPSPITGASGMKMNGPYDYVPPVYWYDKSQKDRGGAWSFNSETSAGVDIPTMDTLKRMMSA
SELDTMWKNPSAKQYHRSSSDTFGNLKLFGDALTKRYGASANLNDFVRKAQLSQYENVRAEFESHSRNYT
DSTNPSTGLIYWMLNSPWTSLHWQLFDAYMDQNGAYYGAKKANEPLHIQYSHDNRSVVVINQTSNAVSGL
TATTKLYNLDGTEKYSNTKTGLSVGALGAKATAVTVPAVSGLSTTYLAKWVLTDSSGKEVSRNVYWLSTK
ADTLNWGGSDWYYTPQSAFADLSGLNNLGQSAVGATANSVAGADGTTTTTVTLKNTSGGRLPAFYVDSKV
VDSAGKPVLPVEWNANAVSLWPGETTTLTAKYRTADLKGSKPSVRISGWNTGTQTVPADGSGPGPSDPVD
YQAEDATIVQGAVESNHAGYTGTGFVNYDNVAGSSVEWTVTVPSAGTYDVVVRYANGTTTSRPLDFSVNG
SISASGVAFGSTGTWPAWTTKTVRVTLAAGVNKIKAVATTANGGPNVDKITL

>CsS
MFHRPASVRRFVTTAVALGLLSTLSTGARAGARTHEPPPRPTTVSSTAGSTTALTGYAIQSTAKVTDPAA
AVSSPGYPASGWYPAGARSTVLAALLAGGKYADPFYSTNQQKIPKADFQVPWWYRSDFTVADTSARTYLD
FSGVISAADVFVNGRQIARSADVAGAYTRHELDVTSLVREGANTVAFRIQPNNPNKNLTMGWIDWLEPPP
DQNMGIVRDVLVRRGGPVALRDAHVITRLDVPSLATADLTVKARARNDSDAAITATVSGSVGATSFRRSV
ALAAHETKTVTFTPADTPGLHLTSPRVWWPAGMGGQPLYALDLSASVSETVSDTVHESFGIRDVKAPLNS
DGARQYSVNGRRLLIKGGGWSPDEFLRWDSTYVEDRLRYALDLGLNTIRLEGHIEPDEFFDLADRYGILT
LPGWECCNKWEGNVNGSGSGDEWTAADYPVAKASMAAEAARLRDHPSVVSFLIGSDFAPDAKIEKTYLDA
LKAADWPTPVVAAASDKSSPVSGSSGMKMTGPYDWIPPNYWYAKREGGATGFNSETSAGPDIPTLDTLRR
MMTPAELDTLWKNPGAKQYHRSPSSVFGTLKIYDAALAGRYGAPTGLTDYVRKAQLAQYENVRAQFEAYG
RGATDASKPATGVIYWMFNSGWTSLHWQLLDRYLDQGGAYFGAKKANEPLHVQYSYDDRSVVVVNNRPAA
VSGLTARVTLFNTDGTQKYDKSATGLSVAGDGAHSTALTLPSSVSGLSTTYLARLVLTDSAGKEVSRNVY
WLSTRPDTLDWAHTDWYYTPTTSYADLKGLGSMARVPVSATASTTAGTDGASTTTVTVRNTGSGRTPSLF
TDVHLVDSKGKPVLPVQWSDNEVSLWPGESATLTVTYRTADLHGSAPRVRVSGWNTAEQTVPAA

>CsG
MIAFLLFLVGTAVATNSKPLATDFGKQDAIPNWDIQQVNKVAGNTKSLSLPNVDTSSWHHAPVSRCTLMA
CLLAEGTYKLDGRDGLWYSDNLAHFDDSLFHVPWVYRNQFDLATKDKSGDVHYFLQTNGITPAADLYLNG
KQIADNVTQSGSYGGHTYDITPLVDKKNALVVKVYPTDYQYDFAVGFVDWNPYPPDNGTGVWRDITIKKT
GNVFMGPVSVLVDMDTPVKKIRNHYAIITVKSQARNLKESPVKFVAKANIKSPDGKLLTVLRKTITLGAG
ESTTVELSTKVQNPEIWWPAAWGEQPLYSAQLTYSIGHDTSDVSPVTNFGIRTVTSELNSHNDTMFSVNG
YPIQITGGGYTSDMFLRWDPRRFEDIARYTLDMGMNTIRLEGKMEQPELYDIADKLGLLLMAGWECCDRW
ESWPYNHDLSMDPPPLWYDDDYKTVNASMRHEAAMMQHHPSVIAFLVGSDYWPDDRATKIYLDGLRDAGW
QVPIIASAAKRGYPKALGPSGMKMEGPYDWVPPVYWSDSEPTDERYGSSFGFGSELGAGVGTPTKGSLGK
FLTNKDMEDLWKKPNKGLYHMSQDTSQFHDRSIYNKALFKRYGKPTSLDDYLTSAQMMDYEAVRSEFEGY
GSQWSASRPATGIIYWMLNNAWPSLHWNQFDYYLHPAGTYFGTKIASRVEHVTFDYFNNFIWVINRSLKK
SGPRSVHIKLVDLKGKEMAQQTMRLSTEPNKSQKVGDISSAIKKIEDVGVLRMTLVDDDSDAILSRNAYW
LSKQKDVVNWTSTTWYYSDVKQYADFTALNKMDQATITLKHSAGKTAGKWNIRVKNTSPVPAVFVQLNLV
DRKGNDVVPLTWSDNYFTLLPLEEIEVEVVDRSAKGAVVEMSGKNIKASRTRLH

>CsM
MERGSDHDTPDTKAEEVPPVSSEAVLPDASHATFRAKLAHHKWVITAMVLVLTAALVTGLAVGLTRGHGY
GYGSANMIGTAMGLAAPVPAWRIQSTAQAGNDLSVLSKSGADVSSWYQIDVSKCTLMACLIQAGVYNDTD
LFFSDNLKKVDASRFSVPWIYRQEFSLDAAAVASGSGNHFFLQTEGITPKGDVWFNGNQLADKTEHAGSY
VGKRFDITNLVGTNNVLAIRVYPTNYNYDFALGFVDWNPYPADNGTGVWRDVTVHQTRGASVTDYRVVTG
MASPSGQGGGNVTLKAVVTNLENQRATIDVQGLVTCNSSTSNKYEGQQSLDIGPLESREFSLTIPVQDAR
IWWPKTWGAQPLYSAHLSVSVKGELADSSERTFGFRTVTSQVNKFNDTIFYVNGQPFQVVGGGYSADMFL
RWDPAKFEAQARYMLDLGHNTVRLEGKQEHPELYDIADRLGLMVMAGWECCDKWEAWSYNQDLAIQPPPV
YSDADYNIAAASMRHEAMMMQSHPSMLTFLVGSDYWPDDRATKLYVDAFKAADWQLPIIASASKRGYPKL
LGPSGMKMEGPYDWVPPNYWYDTDDSKNRKGAAFGFGSELGAGVGTPEKGSLRKFLSPKDMDDLWKQPKK
GLYHMSNEGSQFFTREIYNEALVNRQGGGSGQGSAMADSLDDYLLKAQMMDYEATRAEFEGFAARWNAER
PATGLIYWMLNNAWPSLHWNLFDYYLHPAGSYFGAKVGARTEHVAYDYVKGSVYVINKSLDKRGTRNVAV
QIISLDGKLISSAGGKVDTEPNKSKVVASLASAIRGIKDVVLVRLTLADDKNTTLSRNVYWLANGTDTLD
WSDSTWYHTPVTKYADFTALNKMQEAEVQVSTKASAKGNGARTVIVENKATVPAVFVRLNLVDATGADVN
PVIWSDNYVTLWPGETLELEVTEGADKGKSIEVSGKNVASITVAFD

>CsN
MHLRRSPVTLQVLFGAILGFAIATAADSSSALVSTPGQVAPVPAWYLQSSTKAASDLEALSRPGIDTSGW
LHVNTSKCTLMGCLLEAGIYNDTELFYSDNLRRVDSKQFEVPWLFRQEFGLDPGAGKYFHLLTHGITSRA
DVYLNGKQVADKKTQAGSYGGHTYDITDLVDKDNALLIQAYPTSYYADLAVGWGDWNPWPADNGTGVWRD
VEVKQTGSVALGPLRVVTQLGTPLDGSPANVTLKSRAQNLENIDVTITVTGTVSPEGYADQSIIWSQTVT
LPPLSTTDVTLTTTVQKPAIWWPKQWGSQPLYNAQLSVFASNNTLSDQVSAVFGFRTVTSQLNSFNDITF
LINSHPFQVLGAGYAPDMFLRFSPAKFESEARYVLDLGFNTIRLEGKNEHPELYQIADRLGIMILAGWEC
CDKWEAWSYNQDLTVPTPVWSEDDYNIANASMFHEAGMLQTHPSILGYLIGSDYWPDSKAAPMYINTLRL
QDWQTPVLSSASKRGFPSFMGSPGLKMEGPYDWVPPNYWYDTEPSAGRFGAAFGFGSELGAGVGTPDSSS
LHKFLSPSDLTDLWKNPNKDLFHMSSTETSSFRNRRIYNTGLWNRWSAPTSLEDYVQKSQITDYEATRAQ
FEGYAANWGNSQRPATGMIYWMLNGAFPSLHWSIWDYYMHPAGAYFGAKVGSRIEHVAFDYVKKSVVLIN
RSLDKEGPRSVDIEIIDPTGKTLYTKTVKTTTEPNTSREITSLASAFEDIKDVVFLRLVLSGTSPKDSDT
SPKNSNPSSDVLSNPSSSSSKNTELKALSNPSSKASNPSSKILFNPSSSSKNTELKALSNPSSKASNPSS
DVLSNPSSSSNANPSSSSSSNPSSSARNTEHKVLSSNTYWLSSTLDTLSWDNSNWYYTPVSEYSDYTALN
KLPAANVSVSVTALSKGHSEYESSKVQVTLENHSSFPAFFISLNLVNKEGQDVVPVIWDDNYLTLWPREK
ATVQVGTLEGAELVHAAAAVKVAGKNLKGMTVMVG

>CsAn
MALRNRIVMPGWHIQNTEVLQGPISGWSLPNADVSNWSRIGSKGTVMGGLIESGRYNEQTLFRSDTLSTI
EKSHFQSPWLFREEFSIPELDAGHHVFLNLHGVSSKADVFINGHLVVSCEDQKGAYAGRTQEVTAFIRPE
VNCLLIQAWPTDYLKDMAISFADWNPAPPDNGMGVWRHVVIKVTGPVSVSPLRVTYAQDEANPHSRVTVT
VKTEIENHENAPQDVAIHCQIAVPGNSQNVALSRRMYLLPHSRSTVALETTLEDDQVQIWWPAAWGQQPL
YRVDLNVSLPPSFISDSAWAGFGIRKVEYTLNNHGDGQFFINGRHFQVRGAGYAPDIFLRFDPERVEAIL
RYAVHIGLNTIRLEGKLENSILYGLADRLGLLVMAGWECCDKWEGWTYNEDVDGVEVWKDEDYRIGEASM
LHEAEMMQTHSCMLAFLVGSDYWPDERATAAYLGALNRMDWSNPVIASASKRGHPPQLSSSGMKMEGPYD
WVPPGYWWGERLGAAFGFASELSAGSGTPELSSLRRFLSDVDLASLWGKPQAGHYHQAPSGSVFHSREIY
NKALKRRYGSPTSLEDYVFKCQVMDYEATRAQFEAFAARQNAARPAAGVVYWMLNSAWPSLHWQLFDYYL
YPMGAYYGAKIGARPEHVAFDYHSSSVYLINHSLSAGVRRITAECIDLRGRRLYHREETVEATEITSKQV
FSIPEVTTLATVALMRLVLSASDVEISRNVYWLNGRMDRLNWDKSTWYYTPTTRYADFTSLSQLEQAKMN
TSIDMLAHPDPSMKQLRVRLQNMSSIPAFFVRLTVHDPMTGEQLTLYYTNDSSAYSPVCPTLQIGHHQQK
TINNATMAQTATLVSEILQSSPLDLVPYENLYKHFHANPELSLQEQRTSQRIVDHLASLNAYDIHTGIGG
FGLAGVLRNGPGKTVLLRADMDALPVKEETGLPYSSTATATDPDGVSRPVMHACGHDMHITALLAAAEQL
VRVRDKWNGTLVVLFQPNEERGAGAQAMVDDGLYDKIPVPDIVLGQHVMRLRAGTINCRTGTIMAAADSM
KITVFGRGGHGSLPHTTVDPVLLAAHIVVRLQGVISRELDPDDVGVLTVGSLQAGQTENVISDKAEIGID
FRSVSLETREKIVGAVKRIVKAECMASGSPKDPIFTPTRRFPPTVNEIALASKLAGSFSAHFGADFDPDT
PRTMVAEDFSILATACGRPSCFWFLGGIDRDIWDALVKDGKEGEIPGNHSARFAPVIQPTLKVGAEALAV
GALTFLCRED

>CsH
MLANAIAALLLGSGIASAAGHGSPLTSKAGHRAVIPDWDLKSSSDVSKDLGGLSKPGVDTSSWYHAGTSR
CTIMGCLINAGVYNDEELWYSDNLNHVNWGQFSVPWVYRHEFALSPAKGKHFLLQTNGITSKADLFFNGK
QIADKEYQSGAYAGRTYDITNLAAKKNALLVQVYPTDYLYDFALGYVDWNPYPSDNGSGIWRDITIKETG
SVSMGPVSVLVDIDVPVEKNPARVTVRAEAQNLESHAVEFNAAAVISGNSCSGEALKQTIKLAPGEKKLV
QFTQTIKTPSIWWPKQWGDQPLYTAEVTFSVKGAVSDTAQTKFGVRKVTSFVNQFNDTQYSVNGHPFQVI
GGGYGADMFLRWDSERFTRIVEYMLDMHQNTIRLEGKMEHPELYEICDEYGLMVMPGWECCDKWEAWAYN
DELAIFPPPVWDANDYETANYSMIHEAAMMQPHPSVLTFLVGSDFWPNDEAVVLYANALKNAGWQTPIIA
SASKRGFPALLGPGGMKMDGPYDWVPPNYWYDVEPSEDRLGAAFGFGSELGAGVGTPELSSLRRFLNQSD
LDDLWKNPNKNLFHMSTNASSFYNRKIYNQGLWKRYGAPTSLDDYLLKAQMMDYEATRAQYEGFGALWTA
SRPATGVIYWMLNNAWPSLHWNQFDYYLHPAGSYFGTKVGSRIEHVAYNYQKKEIWVINHSLYQTGSRNI
KVELIDMNGKQIAKKLVQVRTKANSGFKAMDISSDINKLSSVAFLRLVLSDEKGSVLSRNVYWVTKTIDE
LNWDESTWYYTPVSKFVDYTPLNTLATAQVSVTTSGGKHLPGIPGSQTRTVTLENKSSVPAVFIRLTLVD
SKGNDVNPVSWSDNYVTLWPHEKLQLEVGGWDGSGDKIQISGKNIKATTVKL

>CsE
MDWIILLYGLYVAAFGVTVVDGIGETAGDTTTLSQWRLQSSLAVSNVSAWSLPGNDDSSWYQVGARATVM
AGLLENGVYDDTTLFYSNNMETKTGDAAKFDAPWLYRHELLIKNKIRQGEHYLLHTHGITSKADIYVNGV
LVAPSSVQQGSYGGHTYDITKYLRVDANALLIQAYPTNYLRDFAQGFVDWNPYPADNGTGLWRDVMISKT
GPVSISSPRVLTDFRKPDGKPVTVNVLVDVTNHEDETVTGTVQGSIQSDTESLVLSQRFSLQPHETKTIP
MTVRIEKPKVWWPAQWGSQPLYTVNVNATVGKDQVSDVALPRRFGIRHVDSHVNSHNDTAFTVNGSPFLV
LGGGYSPDLFLRFDATRVRNIFQYMLDMGLNTVRLEGKQEHPELYDLADEMGLMVIAGWECCDKWEGWDY
NDEASGEKWTEKDYPIANASMLHEAAMMQTHPSMLAFLIGSDFWPNDRATKIYLDALHRMDWPVPIIASA
SKRGYPKVLGPSGMKMDGPYDWVPPNYFYGDQLGAAFGFGSEEGAGVGTPELTSLRKFLSPKDLDSLWKN
PDQNQYHMSRYDSSFYNRALYNKALFARYGAPGSLEDYLLKVQMMDYEATRAEFEAFSVRQNASRPATGV
IYWMLNSAWPNLHWQLFDYYLSPAGAYFGTKIGGRMEHVAYDYEERAVYLINRSLEKKGPRIVSVDLVDR
HGKSLFNQEVTIDTTPTASKRVIPVKAVTQIKDVAFLRLVLKNPSTGAILSRNVYWLSAKNDVLDWENSD
WYYTPVTKYVDYKALISMPTAAVTASLKRLPAKDGLSQVQVVLKNPSATPAVFMHLSAINKDTQEEITPV
FWSDNYVTVFKGESVTLTAAFPGGRSNWEVILSGANVKRTLLS

>UnA
MRTYLPGESEEVLSEGWTLTLTPADACETPSDIPASLESLHVSVPGTVGQALEAAGKFDRLAPVALNDRD
AWYRLTMISDARERAILRFDGLSTIAEIFFNGELIAASQSMFERLEVPVQMTGADELSICFRALAPRLEK
PGPRARWRPQMMNTQGLRLIRTTALGYMPGWCPEIHSAGPWRPISLIKQDGVLCTLRSSLDDTGTGKVSI
VLQANRDIQSARLICAGYSINLSKAEDGKLLGELIIPGVEIWWPHTHGRPALHEVVLELDGNQHRLGRTG
FRQVEIDHGEDGNGFGLKVNGQPVFCRGAVWTTADIVRLPGTRPDYEPWLKKAAEAGMNMIRVGGTMAYE
TPEFFALCDELGIMVWQDAMLANFDYPAKDDGLRQHIVMEIEQLLESTALSPSFAIFCGGSEMYQQGAML
GLPEQIWKGTLTEEILPELVAEKRPDAAYVANSPSGGALPFFPNAGVGHYYGVGAYCRPLEDARRADLRF
AAECLAFANIPEQETLDRYLPGVAVHDPRWKARTPRDRGASWDFEDVRDHYLKLLYEAEPDVLRREDGAL
YLDMSRAATAEVMEATFAEWRRSGSSCQGALVWTLQDLVPGAGWGIIDAAGRPKSVWHALKRAFRPVQVS
LSDEGTNGLDIHILNDTGDSLNTMLELTCLREGLQPVVHAKRPLALLPRESQTIAATDLFGAFFDTTYAY
RFGPPSHDVTIARLRDIATGHVIADAFHFPLGRKKALHAANLQVAISETNGHWSLEIGTDRLAQSVHITA
EGYRACESWFHLGPGEARKIGLLPETATTEAPPSGEVVSLGSSRRFSF

>UnBv
MLATAAGAAREPRDLPVAGGGWIPATVPGTVAQALALAGRLDEAPSLDERDHWYRIELRGHGPRVLRFHG
LATLAQAWLDDTPILHSDSMFVAHDVSVSLDGTHRLTLCFRALAPHLRDTRAPRRARWRTRLAEPAALRT
IRTSLFGHMPGWFPPHVPTGPWRPVDVLDPADGPVLVDCDLHARIEGDTGWLDAELTFAAPLPDALAARL
SCGKHHATLERAGADRLRASVAVPNVCRWWPHTHGEPVLYDVALHLGDALRPLGATGFRTIEVDAGADGK
GFGLRINGVPVFARGACWSSAAPLALHADDATYARLLGLARDAGFNMIRVGGTMTYEADAFHAWCDRLGL
LVWQDFMFANFDYALDDPAFADNVDREARQFLSRHSASPSLAVLCGGSEIAQQAAMSGLGPKQRFLELTA
DRLAGHAAACRPDVPYVPDSPDGGVLPFAPRERVSHYYGVGAYLRPLDDARRADVRFASECLAFSNVPCD
ATLAELGWPGVHEPRWKAAVPRDPGTSWDFEDIRDHYLQTLYDVVPDRLRREDPSRYFELSRSVIADLMR
ETFSEWRRTGSRCAGALVWQFQDVMPGAGWGMIDAAHRPKSAWHALRQVLQPVQVLLVDEGLNGLDVHVV
NERPAPLSAALELVALRDGRTPVARAGCPVRIAAHDTVRIGSAELLGRFFDWTYAYRFGPCEHDTVVATL
RADDGTLLSQAFHFPSRTHPAVLARRELGLEACVTRTGDTWHVDIDTRHVARHVQIDAPGFMPRDDWFHL
APGTPARVALVPLEMIGKHAAATDGPPAVEIRAVNAARTVRATQAG

>UnBc
MGAQSVRGAAVYDRDGLRGRPVHVLQVSARRAGTTVRHRRAGTLDLRAARPAHRDAGRARPSDDRRGRRV
VPARYARQRLSHAACEDDDRHRRDRPRAPPARLLPQQRLLRGRGDRLRRARRRQLADAVAAVRRVREAAF
RAARRACAVRRVARRLAAASAAPAAGQSGGGVPPPVAGRCEVARRVAARTLPRVLVQFGAAARRELRAVR
SLRALAAGERLHRPVRRHTHGVRAHRGGIDGARIPACARLRARQGGARRCDARRDRSGLYRPDRMRAADQ
PAAAAPGARSLRSGRRAGDAVTRASAMPAWSMLATPAGAAPAPSDLPAGAGWIAAPVPGTVAQALAVAGR
LDEAASLDERDHWYCVELRGHGARLLRFHGLATLAQAWLDDTPILHSDSMFVTHDVRVTLAGQHTLFVCF
RALAPHLRTARAARRARWRTRLAEPATLRTVRTSLLGHMPGWFPHHVPTGPWRPVDVLDPTEGTVPAHCD
LHARVDGDTGWLDAELTFAAPLPDAFAARLSCGEHHATLERAGADRLLATVAVPNVRLWWPHTHGEPVLY
DIALHVGAERIVLGATGFRTIEQHAGDDGNGFGLRVNGTPVFARGACWSSAAPLALHADDATYARLLGLA
RDAGFNMIRVGGTMTYEADAFHAWCDRLGLMVWQDFMFANFDYALDDRAFADAVDAEADQFLARRRASPS
LAVLCGGSEIAQQAAMSGLGPKQRAVELTAERLAARAAAWRPDVPYVSDSPDGGVLPFVPRERVSHYYGV
GAYLRPLDDARRADVRFASECLAFANVPCDATLERIGRPHPHEPRWKAAVPRDPGASWDFDDVRDHYLRT
LYDVAPERLRREDPARYFELSRAVIADVMRETFSEWRRTGSRCAGALVWQFQDVMPGAGWGLLDAAHLPK
SGWYALRQVLQPVQIVVVDEGLNGLDVHVINERPAPLTASVELVALRDGRTPVARCGGQVRLAAHDAVRL
GSAELLGRFFDWTYAYRFGPCEHDTVVASLRGDDGALLSQAFHFPSRTHPAVFARRDPGIEARVSRAGDA
WHVDIDTRHVARHVQIDAPGFMPLDDWFHLAPGATARVALIPQPIGTPRIDDAPPSVDIRAVNAARTVRA
ALAV

>UnBm
MKSAPDRVARGAAQWTLIATPAGAIARPSELGEAGWCAASVPGTVAQALAAARRFDPAHPYPLGDSDYWY
RTTLHGAGPHIVRLNGLATIAEVWLDDTLLLCSDNMYVAHDLPVTLGGAHRLALCFRSLDRHLAEHPPRG
RARWRTRLVDTPALRGVRATFLGRMPGWFPAIEPVGPWRPIDIVNPAGAPTIVHDTLRATLDGRDGVLDA
TLEFAAPLPRTARAQLVCGEHAAPLEATGPRTARATLRIANVTPWWPHTHGEPALYDVGVAIGGATIALA
KTGFRTLAVERGADGRGFALSVNGTPLFARGACWTSADPVGLHADAPAYRRALVLARDAGCNMIRVGGTM
IYEADAFYALCDELGLLVWQDFMLANFDYPSNDPRFAESLKREAEQFLGRHMARPSIAVLCGGSEIAQQA
AMVGLAPDERRVPATEQWLAELCAAHRPDAAYVSDSPHGGVLPFAPREGVTHYYGVGAYLRPPEDARRAG
VRFASECLAFANVPCDATLASIGSPAAHEPAWKRAVPRDPGAPWDFDDVRDHYLRALYGVEPARLRSIDP
ARYLTLSRAVVADLVGETLAEWRRVGSSCAGALVWQFQDVMPGAGWGLVDAHGRPKSAWHALRRVSQPRQ
ILLTDEGLNGLDVHVLNDAPAPLEARIELVALRDGKTPIARAARTVHVAAHAGQCVNSADLLGRFFDFTY
AYRFGPREHDVVIASLYASDGALLSQAFHFPERTAPTVFERGDIGLEASAAYRDGRWCVQVQTRTFARYV
HVCAPGLLPDIDWFHLAPGAAARIEFAADPHSPAPDHRPPEADAAHCAPPAIEVRALNSNKTIRPRIEN

>UnBp
MKSAPDRVARGAAQWTLIATPAGAIARPSELGEAGWCAASVPGTVAQALAAARRFDPAHPYPLGDSDYWY
RTTLHGAGPRIVRLNGLATIAEVWLDDTLLLCSDNMYVAHDLPVTLGGAHRLALCFRSLDRHLAEHPPRG
RARWRTRLVDTPALRGVRATFLGRMPGWFPAIEPVGPWRPIDIVNPAGAPTIVHDTLRATLDGRDGVLDA
TLEFAAPLSRTARAQLVCGEHAAPLEATGPRTARATLRIANVTPWWPHTHGEPALYDVGVAIGGATIALA
KTGFRTLAVERGADGRGFALSVNGTPLFARGACWTSADPVGLHADAPAYRRALVLARDAGCNMIRVGGTM
IYEADAFYALCDELGLLVWQDFMLANFDYPSNDPRFAESLKREAEQFLGRHMARPSIAVLCGGSEIAQQA
AMVGLAPDERRVPATEQWLAELCAAHRPDAAYVSDSPHGGVLPFAPREGVTHYYGVGAYLRPPEDARRAG
VRFASECLAFANVPCDATLASIGSPAAHEPAWKRAVPRDPGAPWDFDDVRDHYLRALYGVEPARLRSIDP
ARYLTLSRAVVADLVGETLAEWRRVGSSCAGALVWQFQDVMPGAGWGLVDAHGRPKSAWHALRRVSQPRQ
ILLTDEGLNGLDVHVLNDAPAPLEARIELVALRDGKTPIARAARTVHVAAHAGQCVNSADLLGRFFDFTY
AYRFGPREHDVVIASLYASDGALLSQAFHFPERTAPTVFERGDIGLEASAAYRDGRWCVQVQTRTFARYV
HVCAPGLLPDIDWFHLAPGAAARIEFAADPHSPAPDHRPPEADAAHCAPPAIEVRALNSNKTIRPRIEN

>UnR
MKLQTCLDASLAAPGRQSPRIERPGIAVVDAALQGEWTWTQTPPGAADSPAALSPGLAWLPAPVPGTVAS
ALRAAGRWDDAAPLPLHVHDHWYRVVFTGDGRRVLRFNGLATLAEVWLNGRLVLSTRHMFVAHEVEVDLA
GSNLLHLCFRALDPWLRRQRGPVRWKPRMIVPPQLRTVRATLLGHMPGWCPPVHAVGPWRPVELLDPTGS
DILHGLRADLWARLHEQDGEVSVRLHFAASAPATGAVELSDEAGCQGQAALVRIDRHTLEGTIHVERPRL
WWPHTHGEPSLYRVDATIDGRSIPCGRVGFRSVSTECPAEAGAFTLRINGERLFCRGACFSSIDLTGLAD
TDQASRRWLELARAGGMNMVRISGVTCYPGEAFYRNCDELGLLVWQDFMFANFDYGSIGPGVGLTEDATR
EVGEWLASTRAHPSIAVACGGSEAEQQAAMLGTSRSEWKQPLFDTLIPQLVAAHRPDIVYVGNSPNGGAW
PFQPDGGVSHYYGVGAYERPLADARLAQVRFASECLAFSNVPCDRTLAEMGTPPLHSPRWKATVPRDAGA
PWDFEDVRDHYLRTLYDVDPSRLRYEQPQRYLILSRAVVAEVMSDVFAEWRRVGSGCAGGLIWQFQDLVP
GAGWGIVDARGRPKSAWHALQQAWQPLQVLITDEGLNGLHLHVLNETPQPRRLLLELCCLRDGEIVVEKV
RHSLRLPAHGAERVEAAALLDRFVDFTYAYRFGPPSHDVVIATLMDENGGEPLSQAVYLPDRRAAALKPP
ELQAQVERVDDDWWLTITARRFARWVHIEDHAYQATENWFHLGPGCSRRVRLMRDSADTSQVDAVPSGEI
YAVNAERSLGYDG
