# Supplementary material for: CLUSS: Clustering of protein sequences based on a new similarity measure
Source: BMC Bioinformatics. 2007 Aug 4;8:286. doi: 10.1186/1471-2105-8-286 (PMC1976428; doi:10.1186/1471-2105-8-286)
Supplement: Additional file 19 — Comparison between the execution times of SMS and ClustalW [file 1471-2105-8-286-S19.pdf]

Comparing execution time of SMS with that of ClustalW

Execution times (i.e., in seconds) obtained by SMS and ClustalW to calculate the pair wise similarity measures of the six randomly generated subsets from the COG database and the G-proteins family. Time is indicated in seconds.

Protein sequence subsets	SMS	ClustalW
SS1 (469 proteins)	98	493
SS2 (743 proteins)	216	1264
SS3 (455 proteins)	107	583
SS4 (409 proteins)	76	449
SS5 (564 proteins)	94	533
SS6 (6444 proteins)	4154	95413
G-proteins (381 proteins)	73	412
